# Homoploid hybrid speciation and recurrent hybridization along the northwestern Iberian mountain chains

**DOI:** 10.1093/aob/mcaf086

**Published:** 2025-05-05

**Authors:** David Criado-Ruiz, Irene Villa-Machío, Rosalía Piñeiro, Jonathan F Wendel, Gonzalo Nieto Feliner

**Affiliations:** Real Jardín Botánico (RJB) CSIC, Madrid 28014, Spain; Departamento de Biología, Facultad de Ciencias, Universidad Autónoma de Madrid, Madrid 28049, Spain; Real Jardín Botánico (RJB) CSIC, Madrid 28014, Spain; Evolutionary Biology Research Group (GIBE), Interdisciplinary Centre of Chemistry and Biology (CICA), Universidade da Coruña, Campus da Zapateira sn., A Coruña 15071, Spain; Department of Ecology, Evolution, and Organismal Biology, Iowa State University, Ames, IA, USA; Real Jardín Botánico (RJB) CSIC, Madrid 28014, Spain

**Keywords:** Cytonuclear discordance, climate-driven range shift, genomics, homoploid hybrid species, *Phalacrocarpum*, reticulate scenarios, spatiotemporal context

## Abstract

**Background and Aims:**

Natural hybridization can lead to diversification and adaptive introgression, among other outcomes. However, the mechanistic underpinnings of this process are insufficiently understood. A previous study of the Iberian endemic diploid genus *Phalacrocarpum* (Asteraceae, Anthemideae) identified several homoploid hybridization events and cryptic diversity, but raised questions regarding their evolutionary significance and specific genetic groups involved. This study aimed to clarify the evolutionary history of this genus.

**Methods:**

A double-digest restriction-site-associated DNA sequencing approach was used to generate nuclear single nucleotide polymorphisms (SNPs) from 261 samples of this genus and plastid sequences were obtained from 121 samples by genome skimming. Phylogenomic relationships were inferred from each of these two datasets. Bayesian genomic clustering analysis and ABBA–BABA tests were used to explore the population genetic structure and specific introgression hypotheses.

**Key Results:**

Bayesian clustering analyses identified *K* = 34 as the best partition for the nuclear SNP data although about half of these groups are minimally represented in each sample and are shared across taxa. The suboptimal partition *K* = 7 supports the existence of seven lineages. In the nuclear phylogenomic tree, *P. oppositifolium* subsp. *anomalum* and subsp. *hoffmannseggii* are not monophyletic. The latter and the western group of subsp. *anomalum* consist of several small clades, each showing varying degrees of admixture. Comparison with the plastome tree reveals cytonuclear conflict affecting mainly two taxa, suggesting historical range shifts and different hybrid origins. ABBA–BABA tests found evidence of introgression, supporting the hybrid origin of the Galician–Portuguese border group, and multiple hybridization events between *P. oppositifolium* subsp. *anomalum* and subsp. *hoffmannseggii.*

**Conclusions:**

Multiple, partially recurrent, hybridization events, occurring mainly along an L-shaped mountain corridor from the eastern Cantabrian range to the Serra da Estrela in central Portugal, have shaped the genetic structure of *Phalacrocarpum* diversity. A part of these events has resulted in three homoploid hybrid lineages, which are incipient hybrid species.

## INTRODUCTION

Mounting evidence from genetic and genomic studies have demonstrated that natural hybridization is and has been a common phenomenon throughout the evolutionary history of a range of organisms, including plants (Abbott *et al.*, 2016; Payseur and Rieseberg, 2016), animals (Mallet, 2005, 2007; Schwenk *et al.*, 2008; Lamichhaney *et al.*, 2018; Edelman *et al.*, 2019) and fungi (Giraud *et al.*, 2008; Leducq *et al.*, 2016). Hybridization, which encompasses allopolyploidy, is recognized to have played an important role in the evolution of numerous flowering plant lineages (Stebbins, 1950; Soltis and Soltis, 2009). This is because hybridization can provide novel raw genetic variation and phenotypes at a much faster pace than can mutations (Rieseberg and Wendel, 1993; Soltis and Soltis, 1993; Arnold, 1997), thus contributing to diversification and adaptation (Nieto Feliner *et al.*, 2020).

Hybridization outcomes are diverse, including the formation of hybrid zones (Barton and Hewitt, 1985; Thompson, 1994), the introgression of genes or entire organelles from one lineage into another (Rieseberg and Wendel, 1993; Hanemaaijer *et al.*, 2018), and the formation of new species through homoploid or polyploid hybrid speciation (Rieseberg *et al.*, 2003; Cronn and Wendel, 2004; Gompert *et al.*, 2006; Mallet, 2007; Vallejo-Marín and Hiscock, 2016). Additionally, hybridization may reinforce barriers to gene flow, and increase extinction risk through wasted reproductive effort or by blurring the boundaries between lineages (Prentis *et al.*, 2007; Todesco *et al.*, 2016). Despite numerous studies, the intrinsic and extrinsic mechanisms driving hybridization and its consequences remain incompletely understood. Various intrinsic, extrinsic, biotic and abiotic factors are known to facilitate hybrid formation and viability, such as low parental divergence and shared ploidy levels (Tate *et al.*, 2005; Beddows and Rose, 2018; Brown *et al.*, 2023), overlapping parental ranges (Lowry *et al.*, 2008), lifespan and mating systems, particularly outcrossing rates (Ellstrand *et al.*, 1996; Beddows and Rose, 2018). The final landscape of hybridization results from the interplay between these factors and how evolutionary forces such as selection, genetic drift and migration across different timescales and ecological contexts sieve the raw genetic material generated (Abbott *et al.*, 2013; Sætre, 2013).

Exploring how all these factors interact in non-model organisms representing a range of ecological situations and taxa is therefore necessary to gain a broad understanding of the interplay between hybridization and diversification (Abbott *et al.*, 2013; Villa-Machío *et al.*, 2023). However, from an inference standpoint, reconstructing hybridization scenarios becomes challenging, especially when hybridization events are ancient, as defined by Stull *et al.* (2023), or when hybridization has been recurrent (Cronn and Wendel, 2004).

**Fig. 1.**
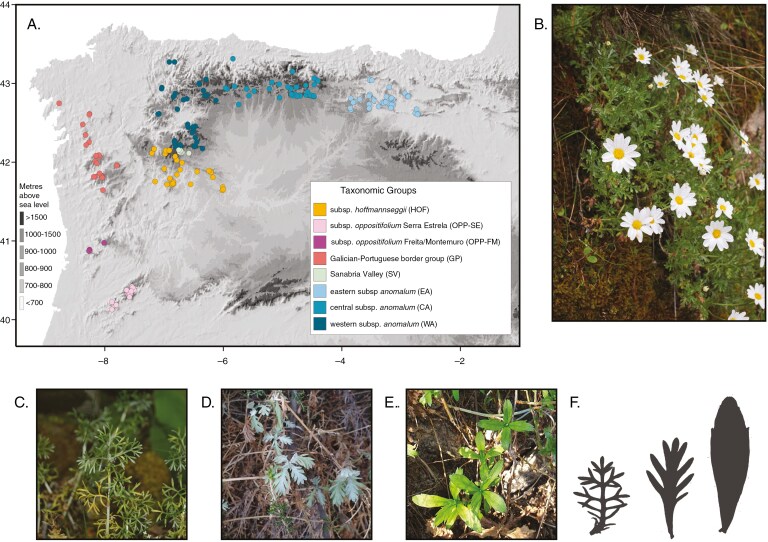
(A) Geographical distribution of the Iberian endemic genus *Phalacrocarpum*. Data are derived from filtered specimen locations obtained from GBIF (https://doi.org/10.15468/dl.am46at), herbarium specimens (MA, SALA, LOU, SANT) and our own georeferenced sampling sites. All locations were verified for accurate taxonomic identification. Colour codes indicate the taxonomic groups considered in this study, as determined by genomic results. (B) *Phalacrocarpum oppositifolium* subsp*. oppositifolium* in its natural habitat in Serra da Estrela, Portugal. (C–E) Leaves of subspecies *anomalum* (C), *oppositifolium* (D) and *hoffmannseggii* (E). (F) Representative leaf silhouettes of these three subspecies.

*Phalacrocarpum* (DC.) Willk. is a diploid genus, 2*n* = 2*x* = 18 (https://taux.evolseq.net/CCDB_web/search/Phalacrocarpum/; accessed 11 February 2025), within the complex daisy tribe Anthemideae (Asteraceae) (Oberprieler *et al.*, 2022; Criado‐Ruiz *et al.*, 2024 ), and a suitable example for studying the consequences of hybridization in non-model systems. It is one of the few (27) vascular plant genera endemic to the Iberian Peninsula (Buira *et al.*, 2017), with a geographical range mostly restricted to the Northwest quadrant. This includes several mountain ranges in Galicia and northern Portugal, extending south to Serra da Estrela, and eastward along the Cantabrian Range up to Álava and La Rioja (Fig. 1) (Nieto Feliner, 1982, 2019).

Taxonomic treatments within *Phalacrocarpum* have been unstable, ranging from recognition of two species and one variety (Nieto Feliner, 1982) to only one species, *P. oppositifolium*, with three subspecies: subsp. *anomalum*, subsp*. oppositifolium* and subsp. *hoffmannseggii* (Nieto Feliner, 2019), which are distinguished by leaf morphology (Fig. 1). Intermediates occurring in two areas (southern Galicia–northern Portugal and Sanabria Valley populations) underlying this taxonomic instability pointed to hybridization and prompted a previous study, based on Sanger sequencing data and a limited pilot study of single nucleotide polymorphisms (SNPs) on 33 samples (Criado-Ruiz *et al.*, 2021). This suggested different evolutionary scenarios underpinning the current diversity in this genus including: (1) recent hybridization resulting in a hybrid zone in the Sanabria Valley (SV); (2) non-recent hybridization events in southern Galicia and northern Portugal leading to homoploid hybrid speciation (GP) and possibly in central Portugal (OPP); and (3) a strong genetic – but not phenotypic – differentiation in eastern Cantabrian populations (a cryptic species), which might have been the result of genetic drift possibly due to isolation and adaptation to limestone substrates (EA). These findings motivated the present study based on more comprehensive sampling and additional analyses in an effort to clarify relationships and history.

Here we investigated the role of hybridization and isolation in shaping the evolutionary history of the genus *Phalacrocarpum*. To achieve this, genomic data – including SNPs derived from double-digest restriction-site-associated DNA sequencing (ddRADseq) and complete plastomes from a genome skimming approach (GSA) – were analysed from a comprehensive sampling of populations across the known range of the species. Analyses focused on hybridization events, examining their context (relative timing inferred from phylogenetic analyses, whether they occurred once or recurrently, and the probable associated range dynamics), to disentangle their effects from those of divergence in isolation, and to shed light on how diversification may have occurred in reticulate scenarios.

## MATERIALS AND METHODS

### Plant material

We carried out an extended sampling throughout the *Phalacrocarpum* distribution range. A total of 217 new *Phalacrocarpum* samples were collected during fieldwork undertaken mostly in 2020, corresponding to 50 different sampling locations. Between one (just one case) and ten individuals were sampled per site to account for taxonomic and population variability. Each sample was initially identified as belonging to one of the six taxonomic groups described in Criado Ruiz *et al.* (2021): *P. oppositifolium* subsp*. oppositifolium* (OPP), eastern populations of *P. oppositifolium* subsp. *anomalum* (EA), western populations of *P. oppositifolium* subsp. *anomalum* (WA), *P. oppositifolium* subsp. *hoffmannseggii* (HOF), populations of uncertain taxonomic assignment from southern Galicia and northern Portugal – termed Orense massifs (OM) in Criado-Ruiz *et al.* (2021), but hereafter called Galician–Portuguese border group (GP) – and Sanabria Valley (SV) (Fig. 1) (Supplementary Data Appendix S1). Four Anthemideae samples were also collected as outgroups for the nuclear phylogenomic analysis (Appendix S1). These include *Leucanthemopsis pallida* and *L. cuneata* subsp. *valdes-bermejoi*, belonging to the same subtribe as *Phalacrocarpum*, Leucanthemopsinidae, and *Chamaemelum nobile* and *Santolina rosmarinifolia*, from the closely related subtribe Santolininae. All four species belong to the Mediterranean Clade (Criado-Ruiz *et al.*, 2024).

In addition to the newly collected samples, those used in our previous study were also part of our dataset once their reproducibility was tested. We selected 11 of the 33 samples previously analysed for inclusion in this new sequencing run (Buono *et al.*, 2021). In the phylogenomic analyses of a dataset containing 11 ‘duplicated’ samples, we compared the position of these replicate samples and verified that differences were minimal (see below). We thus retained the duplicated samples in our downstream analyses.

### DNA extraction and genome sequencing approaches

DNA was extracted from silica gel-dried leaf material using the CTAB method (Doyle and Doyle, 1987) to ensure high recovery of DNA. In some cases, extractions were repeated to achieve the desired purity and integrity. A total of 232 samples were processed (Supplementary Data Appendix S1), including the 217 newly collected *Phalacrocarpum* samples, the four Anthemideae outgroup samples and the 11 *Phalacrocarpum* samples previously analysed by Criado-Ruiz *et al.*, (2021) (see below). DNA concentrations were quantified using 2 µL with the Qubit dsDNA HS Assay Kit (Thermo Fisher Scientific; O’Neill *et al.*, 2011). The purity and integrity of the DNA were assessed using 1 µL with a Nanodrop spectrophotometer.

ddRADseq (Kess *et al.*, 2016), paired-end (2 × 150 bp) DNA libraries were prepared following the same protocol used in the previous study (Criado-Ruiz *et al.*, 2021), based on enzymes Pstl and BglII, and sequenced on an Illumina NovaSeq PE150 platform. This enabled us to include the 33 previously analysed samples in the present study, once the feasibility of combining ddRADseq-based SNP data sequenced at different times was checked (Supplementary Data Appendix S1). Accordingly, the total number of samples in the dataset was 265.

GSA, a method of low-coverage genome shotgun sequencing (McPherson *et al.*, 2013; Male *et al.*, 2014; McKain *et al.*, 2018), was employed to retrieve plastid genomes. A total of 121 *Phalacrocarpum* samples, all of them also sampled for ddRADseq, were selected for sequencing the plastomes (Supplementary Data Appendix S1), ensuring representation of taxonomic and population diversity. Paired-end shotgun libraries were prepared according to the manufacturer’s instructions of the TruSeq Nano DNA High Throughput Library Prep Kit (Illumina) by Macrogen Inc. (Seoul, Korea), and sequenced using the NovaSeq X (Illumina). As outgroups for plastome analyses, we obtained eight Anthemideae samples sourced from the NCBI Sequence Read Archive (SRA), increasing the total number of analysed samples to 128 (Appendix S1).

Demultiplexed reads resulting from both sequencing approaches were trimmed using Trimmomatic v.0.39 (Bolger *et al.*, 2014), removing adapters and low-quality begin/end base pairs (ILLUMINACLIP: TruSeq3-PE-2.fa:2:30:10 LEADING:20 TRAILING:20 SLIDINGWINDOW:4:20 MINLEN:50). Their quality was checked before and after trimming with FastQC (Andrews, 2010). Only paired reads were used for downstream bioinformatic analyses.

### Assembling ddRADseq datasets

In the absence of a closely related reference genome for *Phalacrocarpum*, *de novo* assembly of the ddRADseq data was performed using the ipyrad pipeline (Eaton and Overcast, 2020). This pipeline clusters reads from different samples into ‘loci’ based on sequence similarity, taking several parameters into account. We focused on two key filtering parameters: the clustering threshold (c), which determines the allowable variation among sequences grouped into a locus, and the minimum number of samples (m) in which a locus must be present to be included in the final matrix. Other parameters were set to the default values, including sequencing coverage, mapping quality and those accounting for paralogy, e.g. max_shared_Hs_locus = 0.5 (Eaton and Overcast, 2020).

To construct the *Phalacrocarpum* ddRADseq dataset (261 samples) used in subsequent analyses, except for the nuclear inference (see below), we generated various assemblies, adjusting these parameters to test different levels of sequence variation and sample representation. Specifically, we varied the clustering threshold from 15 % (c85) to 5 % (c95) allowable variation among locus sequences, and the minimum sample presence (m) from 85 % (222 samples) to 30 % (78 samples). We considered the following assemblies representing seven different combinations of these parameters: c85m131, c88m131, c90m131, c95m131, c90m78, c90m183 and c90m222. To choose one assembly, we assessed statistics of the number of loci retained, the number of SNPs and the percentage of missing data (Supplementary Data Fig. S1; Table S1). Additionally, we performed a fast Bayesian genetic clustering analyses with BAPS 6.0 (Corander *et al.*, 2013) to explore the resulting genetic structure and visualized the relationships among the taxa using NeighborNet analyses, based on uncorrected p-distances in SplitsTree 4.10 (Huson and Bryant, 2006). For both analyses, BAPS and NeighborNet, we used unlinked SNPs – one per locus – to avoid over-representation of loci or genomic regions. These analyses enabled us to assess whether the number of inferred genetic groups varied with different assembly parameters and to identify the most frequently observed relationships, respectively (Supplementary Data Material 1).

### Nuclear phylogenomic inference

We applied the polymorphism-aware phylogenetic model (PoMo) (De Maio *et al.*, 2015; Schrempf *et al.*, 2016) implemented in IQ-TREE 2 (Minh *et al.*, 2020) to directly infer the maximum-likelihood (ML) nuclear species tree. Unlike other software such as SNAPP (Bryant *et al.*, 2012), PoMo can handle datasets the size of ours without the need to downsample. PoMo expands classical substitution models by accommodating polymorphic states within populations, making it well-suited for analysing genome-wide data involving multiple species and samples. Furthermore, this method accounts for incomplete lineage sorting (ILS) or hybridization, which may have complicated the phylogenetic signal (Schrempf *et al.*, 2019).

We used all samples, including the four outgroups (265 samples), which required generating a new assembly that incorporated them. To ensure consistency across analyses, we applied the same assembly parameters selected for the *Phalacrocarpum* dataset (Supplementary Data Material 1; Table S1). Vcftools was employed to filter the complete SNP matrix to only include biallelic SNPs present in more than 95 % of individuals (vcftools --max-missing 0.95; reducing from 45 801 to 4258 SNPs). To convert the filtered VCF to the allele frequency input data for PoMo, we first used PGDspider (Lischer and Excoffier, 2012) to get the fasta file, and then the conversion script FastaToCounts.py provided by the python library cfilb (https://github.com/pomo-dev/cflib). A GTR model was selected as the substitution model (-m GTR+P; substitution model with polymorphisms), and 1000 bootstrap replicates (-B 1000 UFBoot2; Thi Hoang *et al.*, 2017) were performed to estimate branch support values. FigTree 1.4.4 (Rambaut, 2018) was used to visualize the resulting trees.

### Population structure and genetic diversity analyses

To estimate the optimal partition of the genetic data under an admixture model, we performed a Bayesian genetic clustering analysis using STRUCTURE v.2.3.4 (Pritchard *et al.*, 2000), also implemented in ipyrad using unlinked SNPs, i.e. one per locus (Eaton and Overcast, 2020), obtained from the selected 261 *Phalacrocarpum* sample assembly. Each run consisted of 1 000 000 replicates with a burn-in period of 100 000. Three runs were carried out for each *K* value (from 1 to 38; number of metapopulations plus 1). This number was obtained by pooling together sampling sites lying within a 10-km radius (Supplementary Data Table S2). The optimum number of genetic clusters was estimated applying the Evanno criterion (Evanno *et al.*, 2005). The replicates from the selected *K* value were aggregated to produce the averaged admixture proportion table. This table was then utilized to generate a stacked barplot using ggplot2 (Wickham *et al.*, 2016) in R (R Core Team, 2020), representing the estimated membership probability of each sample across the inferred clusters.

In addition, to provide an overall picture of the genetic distribution in the chosen assembled dataset, we performed a principal component analysis (PCA). The analysis was conducted both with all the samples included and with the most divergent groups excluded – i.e. the eastern (EA) and central populations of subsp. *anomalum.* The latter was not detected in Criado-Ruiz *et al.* (2021) and is hereafter referred to as CA. We used the ipyrad.pca tool, implemented in ipyrad using unlinked SNPs (Eaton and Overcast, 2020).

### Tests of introgression

Signatures of ancient hybridization (introgression) can be masked in admixture analyses (Lawson *et al.*, 2018; Moran *et al.*, 2021) by processes such as backcrossing, population isolation and diversification (Stull *et al.*, 2023). To search for these, ensuring detection of both recent and more ancient events using phylogenetic frameworks, we performed a series of ABBA–BABA tests, based on Pattersons’s four-taxon D-statistic (Green *et al.*, 2010; Durand *et al.*, 2011), using ipyrad (Eaton and Overcast, 2020). This method provides a metric based on the frequencies of SNPs that are discordant with a hypothesized species tree topology. In a four-taxon bifurcating tree (((P1,P2)P3)P4), with the outgroup/ancestral allele denoted as ‘A’ and the derived allele as ‘B’, the D-statistic evaluates the frequency of two distinct site patterns, ABBA and BABA (Supplementary Data Fig. S2). These patterns indicate instances where an allele is derived in P3 compared to P4 (outgroup) and is also derived in either P1 or P2, but not both. When these patterns result from the sorting of ancestral polymorphisms (ILS), they are expected to happen with similar frequency owing to stochastic processes. When frequencies of ABBA and BABA patterns differ significantly, it is interpreted as introgression between P3 and P1 (BABA) or between P3 and P2 (ABBA) (Eaton *et al.*, 2015).

The four sets of ABBA–BABA tests presented here focused on hypotheses that emerged from the obtained phylogenomic inference based on nuclear SNPs (Table 1). These were consistent with our goal of clarifying the extent and evolutionary significance of hybridization in this genus. The first one (A) investigated the hybrid origin of both the Galician–Portuguese border group (GP) and subsp. *oppositifolium* (OPP) by testing introgression between each of these groups and subsp. *hoffmannseggii* (HOF), the putative parent of both of them (Table 1, A1–3). The second set of tests (B) also investigated the origin of these two groups (GP, OPP), but looked for the second parental lineage, by testing introgression between either group (GP and OPP) and the three subsp. *anomalum* groups (EA, CA, WA; Table 1, B1–6). The third set of tests (C) explored introgression in western subsp. *anomalum* populations (WA) from other subsp. *anomalum* groups (EA, CA; Table 1, C1–2) or populations of the same group (WA; Table 1, C3). This tests the recurrence of introgression between WA and HOF, whereby different WA populations contacting and exchanging gene flow would lead to genetically distinct offspring depending on the degree of introgression by HOF. Note that this hypothesis, using two different WA populations as P2 and P3 (C3), is justified by the staggered pattern of WA in the phylogenomic tree (see Results). The fourth set of tests (D) examined introgression in populations from the Sanabria Valley (SV) by subsp. *anomalum* groups (EA, CA, WA; Table 1, D1–3), which have been suggested to represent recent gene flow in a hybrid zone (Criado-Ruiz *et al.*, 2021). While for A, P4 were different subsp. *anomalum* groups, for the last three sets, P4 grouped the same four species of Anthemideae used in the phylogenomic analyses.

**Table 1.** Set of introgression tests explored using ABBA–BABA. (A) Hybrid origin of the Galician–Portuguese border group (GP) and subsp. *oppositifolium* (OPP) by testing introgression between each of these groups and subsp. *hoffmannseggii* (HOF), the putative parent of both of them. (B) Same hypotheses as A, but focusing on the second parental lineage, by testing introgression between either group (GP and OPP) and the three subsp. *anomalum* groups. (C) Introgression in western subsp. *anomalum* populations (WA) from other subsp. *anomalum* groups. (D) Introgression in populations from the Sanabria valley (SV) by subsp. *anomalum* groups. The total number of tests conducted, the percentage of significant results and the patterns supported are presented. OUT: *Leucanthemopsis pallida*, *L. cuneata* subsp. *valdes-bermejoi*, *Chamaemelum nobile* and *Santolina rosmarinifolia.*

Set of tests	P1	P2	P3	P4	No. of tests	Percentage significant tests	Pattern
A1	OPP	GP	HOF	EA	840	48.33	ABBA
A2	OPP	GP	HOF	CA	840	57.74	ABBA
A3	OPP	GP	HOF	WA	840	23.09	ABBA
B1	HOF	OPP	EA	OUT	540	0.19	BABA
B2	HOF	OPP	CA	OUT	420	0.24	BABA
B3	HOF	OPP	WA	OUT	600	17.5	BABA
B4	HOF	GP	EA	OUT	504	0	–
B5	HOF	GP	CA	OUT	392	0.77	BABA
B6	HOF	GP	WA	OUT	560	8.39	BABA
C1	HOF	WA	EA	OUT	324	0	–
C2	HOF	WA	CA	OUT	252	22.22	ABBA
C3	HOF	WA	WA	OUT	144	93.06	ABBA
D1	HOF	SV	EA	OUT	252	0	–
D2	HOF	SV	CA	OUT	196	3.06	ABBA
D3	HOF	SV	WA	OUT	280	16.79	ABBA

Significance was determined through 1000 bootstrap replicates for each test (Supplementary Data Table S3). Following the tutorial (https://ipyrad.readthedocs.io/en/latest/API-analysis/cookbook-abba-baba.html), all samples were grouped within their own sampling site to calculate allele frequencies, and were used in the multiple combinations of the (((P1,P2)P3)P4) tree. A Z-score was computed to indicate the deviation of the D-statistic from zero in terms of bootstrap standard deviations. Tests were considered significant, and thus indicative of introgression, if the Z-score > 2.58, corresponding to a conservative cutoff of α = 0.01 after applying the Holm–Bonferroni correction for multiple comparisons (Eaton and Ree, 2013).

### Plastome rescue, data matrix assembly and phylogenomic analysis

Plastid gene recovery was conducted with HybPiper v.2.0.1 (Johnson *et al.*, 2016). We prepared a specific plastid target file based on a complete annotated *Leucanthemum virgatum* plastome from NCBI (NC_047461.1), including both genes and intergenic regions. This whole plastome target file, with a total of 209 regions (102 genes and 107 intergenic regions), was used as the mapping template. For each of these regions, unaligned multi-FASTA files were retrieved from paired reads of the 121 *Phalacrocarpum* samples and eight NCBI outgroups, with the *retrieve_sequences* function in Hybpiper. The R (R Core Team, 2020) script *max_overlap* (see https://github.com/keblat/bioinfo-utils/blob/master/docs/advice/scripts/max_overlap.R) was used to identify and discard suboptimal samples and/or regions, building balanced data matrices (Shee *et al.*, 2020). Furthermore, potential paralogues (loci with ≥3 alleles) were identified using the *paralog_retriever* function, and excluded from downstream analyses.

Gene data matrices were aligned using MAFFT v.7.508 (Katoh and Standley, 2013) with the accuracy-oriented algorithm (--genafpair), and alignment summary statistics were calculated with AMAS (Borowiec, 2016). Data matrices were then trimmed to filter poorly aligned regions using trimAl v.1.4.rev15 (Capella-Gutiérrez *et al.*, 2009). The gap threshold (-gt) and the percentage of original data matrix conserved (-cons) were set at different levels to explore their impact on phylogenetic inference: all combinations of -gt 01, 03, 07, 09 and -cons 30, 50, 70. Summary statistics for these trimming schemes were also calculated with AMAS.

For each combination, a concatenated sequence matrix was generated. ML analyses were conducted using the IQ-TREE 2 partitioned analysis (-p) (Chernomor *et al.*, 2016). The substitution model was specified by region. All partition files were generated using AMAS (--part-format nexus). The best substitution model was chosen using ModelFinder, and 1000 bootstrap replicates (UFBoot2; Thi Hoang *et al.*, 2017) were performed to estimate branch support. The programs treespace (Jombart *et al.*, 2017) in R, and FigTree 1.4.4 were used to explore and visualize the resulting topologies. The final plastome tree was obtained by selecting the most represented topology in the treespace landscape with the best support values.

## RESULTS

### ddRADseq data assembly

Sequencing of the 261 *Phalacrocarpum* samples (excluding the four outgroup samples) generated 531 589 839 identified reads, with an average of 2 036 743 reads (± 762 236 SD) per sample. When comparing the first sequencing batch (our previous study) to the new one, the first batch generated a lower average number of reads (Table 2). Based on the criteria described in Supplementary Data Material 1, the c90m131 assembly was selected to generate the dataset used in subsequent analyses. This assembly produced a total of 1699 loci including 43 441 variable sites, 27 936 of them parsimony informative, and 1699 unlinked SNPs.

**Table 2.** Mean and standard deviation of the number of reads obtained for all ddRADseq samples analysed in this study. Numbers for subsets of the full sampling are also provided, i.e. belonging to the first (old) or the second (new) sequencing batch, re-sequenced samples, or outgroups.

Sequenced samples	Number of samples	Average raw reads	Standard deviation (SD)
All samples	265	2 044 647.5	773 066.36
All *Phalacrocarpum* samples	261	2 036 742.7	762 236.47
New samples of *Phalacrocarpum*	217	2 110 187.1	770 340.53
Old samples of *Phalacrocarpum*	33	1 508 402.2	337 343.12
Repeated samples of *Phalacrocarpum*	11	2 172 905.5	771 812.13
New samples of outgroups	4	2 560 439.3	1 370 293.8

### ddRADseq-based networks

For the NeighborNet analyses based on unlinked SNPs, there were some differences in the relative positions of the samples depending on the clustering threshold (c) used (Supplementary Data Fig. S3). However, all networks exhibited a sort of neuron-like shape: two groups of subsp. *anomalum* (eastern and central populations, EA and CA, respectively) at one end (‘the axon’) separated by a long axis from the remaining samples, which appear clustered at the other end (‘the soma’). In the ‘soma’ end, the network contains a number of parallel edges, indicating alternative connections and thus uncertainty, which is consistent with the results of other analyses. By selectively removing groups from the dataset, we gained clearer insights into the relationships within the ‘soma’, particularly between WA, HOF and SV.

For more details on the NeighborNet analyses, both using the full dataset and subsets of it, see Supplementary Data Material 1 and Fig. S4.

### PoMo phylogenomic tree

Hereafter, we consider that a particular node has strong or full support when BS = 100 %, high support when 100 % > BS ≥ 95 %, moderate support when 95 % > BS ≥ 75%, and weak or low support when BS < 75 %.

**Fig. 2.**
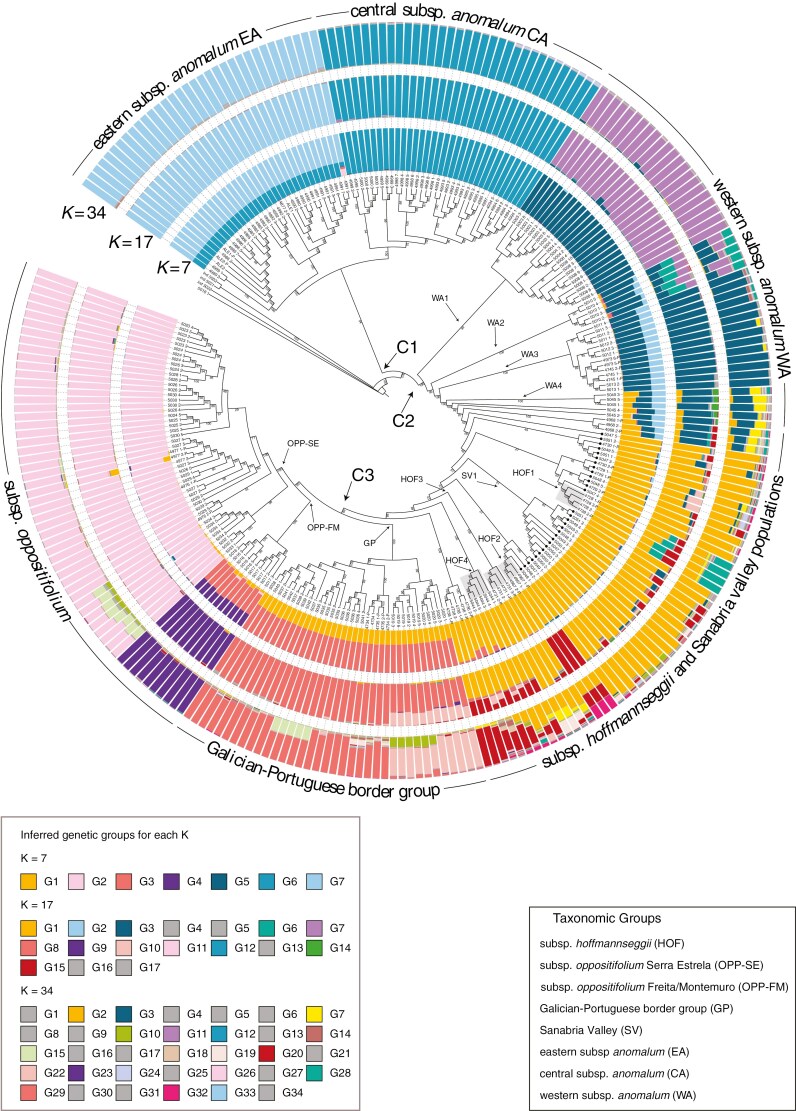
Maximum-likelihood phylogenomic reconstruction of *Phalacrocarpum* using the polymorphism-aware phylogenetic model (PoMo) and Bayesian genomic clustering analysis, both based on SNPs. The three best optimal partitions (*K* = 34, 17, 7) obtained with STRUCTURE are added on concentric rings. Node labels are discussed in the text. Samples with a black circle are from around Lake Sanabria. Sample labels followed by ‘P’ were analysed in Criado-Ruiz *et al.* (2021). Samples with nodal support values (PP) below 75 % are marked in red.

Analysis of the filtered SNP matrix under the phylogenetic model PoMo fully supports the monophyly of *Phalacrocarpum* (Fig. 2). All samples collected within the same population were recovered within their own clade, with few exceptions (see below). Furthermore, the 11 samples that were repeated in the two sequencing sets were recovered as sisters or part of the same clade (sample ALE1 being the only exception).

The recovered phylogeny shows an initial divergence separating two main clades. On one side, a highly supported clade (C1) includes all studied samples of subsp. *anomalum* distributed in the central (CA) and eastern (EA) areas of the Cantabrian mountain range, but not the western samples of this subspecies. The other main clade resulting from the earliest divergence event (C2) has high support; this clade includes the rest of the *Phalacrocarpum* samples. Within C2, the distal portion of the tree encompasses a moderately supported clade (C3) with two monophyletic groups, one (highly supported) corresponding to all subsp*. oppositifolium* (OPP) samples and the other (fully supported) corresponding to all southern Galician–Portuguese border group (GP) samples, except for populations 4736 and 4738. Within the OPP group, two clades are found, each with full support, corresponding to three Portuguese mountain ranges: Serra da Estrela (OPP-SE) and, falling in the same clade, Serra da Freita and Serra de Montemuro (OPP-FM). Unlike the latter two ranges, samples from the same sampling point in Serra da Estrela (4976, 4977, 5024, 5026, 5027, 5029, 5030) are not all monophyletic and nodes generally exhibit lower support. This is probably due to the fact that multiple samples of Serra da Estrela from different localities are part of the same metapopulation that covers a large area compared to those from Freita and Montemuro. The main GP group contains two clades, each with full or high support, and, similar to Serra da Estrela, only samples from Gerês-Xurés (5037, 5038, 5039, 5040, 5041, 5042) belonging to the same sampling point are not monophyletic and exhibit lower support values at internal nodes (Fig. 2).

Excluding C3, clade C2 consists of a large number of subsequently diverging clades of different size, where neither the subsp. *anomalum* samples from the westernmost part of the Cantabrian mountain range and Montes de León (WA), nor the subsp. *hoffmannseggii* (HOF) samples form monophyletic groups. Specifically, WA samples are divided into three fully supported clades (WA1, WA2, WA3) and a fourth group (WA4) that includes most, but not all, samples from population 5045. Within clade WA3, the samples from 4745 and 4973 populations do not cluster in their own monophyletic group (Fig. 2).

*Phalacrocarpum oppositifolium* subsp. *hoffmannseggii* (HOF) samples appear intermingled with those of the conflicting Sanabria Valley populations (SV), although they group into fully/highly supported clades (HOF1, HOF2, HOF3, HOF4). In contrast, SV samples from the same location do not cluster in their own monophyletic groups. Two of these clades (HOF1, HOF2) are embedded in clades that include samples from the Sanabria Valley (SV). All these SV-HOF clades splitting off along the backbone of the tree lack strong support. Regarding SV, only one population (Robleda-Cervantes, 4968) shows strong support. The rest do not form monophyletic groups, or if they do (SV1), with moderate support, they do not include all samples studied – 4729, 4730, 5047, 5048, 5049, 5050 and 5051 (Fig. 2).

### Population structure and genetic diversity analyses

The STRUCTURE analysis, guided by the Evanno criterion, identified 34 genetic groups (*K* = 34) as the best data partition, with suboptimal partitions at *K* = 17 and *K* = 7 (Fig. 2). For *K* = 7, the easiest partition to interpret, group G6 (ocean blue) defines all CA samples and is present also in EA (~33 %) admixed with G7 (light blue). G7 also appears in part of WA and in one Sanabria Valley (SV) population. Group G1 (orange) is present in HOF, SV (~94 and 92 % respectively), GP samples (~33 %), WA (~4 %, but 28 % in 5045) and OPP-FM (~6 %). OPP-FM has its own genetic group (G4, dark violet) together with a significant presence of G3 (coral pink), which also characterizes GP samples. OPP-SE is dominated by G2 (pink), while G5 (dark blue) is prevalent in WA and, at a smaller percentage, in SV (Supplementary Data Table S4).

In the *K* = 17 and *K* = 34 partitions, the main difference with *K* = 7 is that the new genetic groups are mostly represented in taxa that occupy internal/intermediate positions along the nuclear phylogeny and the geographical range of the genus, i.e. WA, HOF, SV and to a lesser extent GP. The SV populations, in particular, show admixture from various genetic groups, none of which are exclusive. In contrast, CA, EA, OPP-FM and OPP-SE remain well characterized genetically although with some admixture in some samples of OPP-SE (5032, 5033, 5034). Also, in these *K* = 17 and *K* = 34 partitions many of the groups are shared across populations, with low representation (<10 %; grey in Fig. 2). Other groups appear in higher ancestry proportions, particularly in HOF, whose main group (orange) (G1 in *K* = 17, G2 in *K* = 34) is present also in GP, SV and WA.

**Fig. 3.**
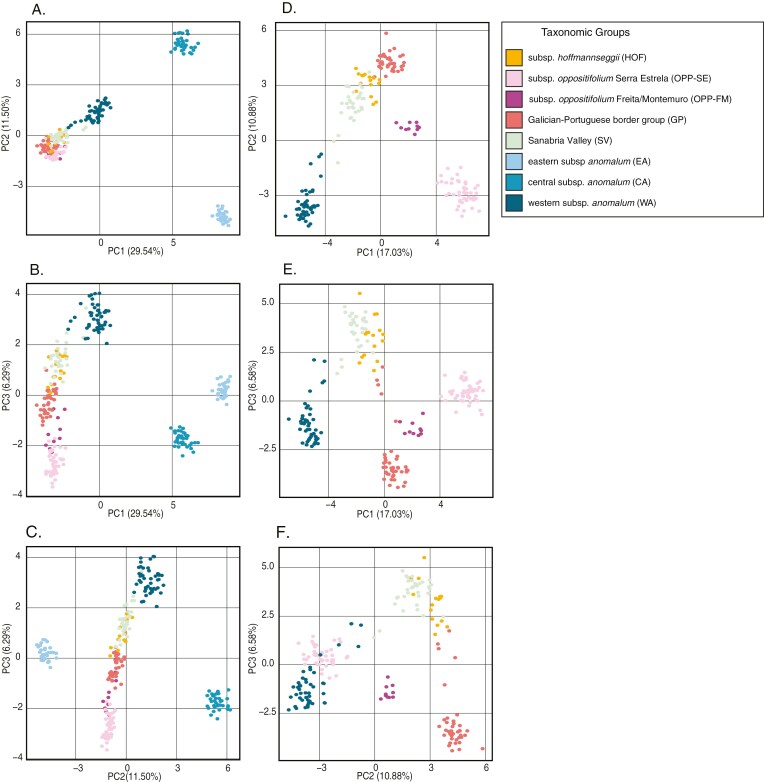
Principal component analysis of *Phalacrocarpum* based on 1699 unlinked SNPs labelled by taxonomic and genetic groups considered in this study, including all samples (A–C), and excluding eastern and central subsp. *anomalum* samples (D–F). A and D include scatterplots of PC1 vs. PC2. B and E include scatterplots of PC1 vs. PC3. C and F include scatterplots of PC2 vs. PC3. The percentage of variance explained by each principal component is indicated in parentheses.

In the PCA based on independent SNPs, the first two principal components explain 41 % of the total variance (29.5 % and 11.5 % respectively). Consistent with the nuclear tree, the scatterplot of the first two PC axes neatly separates three clusters (Fig. 3). Subspecies *anomalum* is split into three groups, with EA and CA standing apart along PC1, and WA clusters with HOF, OPP, GP and SV (Fig. 3A). In the PC1 vs. PC3 and PC2 vs. PC3 scatterplots, one of these three clusters is more diffuse, allowing some spatial differentiation for WA, OPP and GP although SV appears intermingled with HOF. When EA and CA are excluded, the relative position of the remaining groups is more clearly depicted (Fig. 3D–F). For instance, in the scatterplot of PC1 against PC2, WA, GP and OPP-SE are located in the vertices of a triangle, HOF and SV remain intermingled, and the two subsp. *oppositifolium* groups are separated, with OPP-FM in an intermediate position between OPP-SE and GP.

### ABBA–BABA tests

**Fig. 4.**
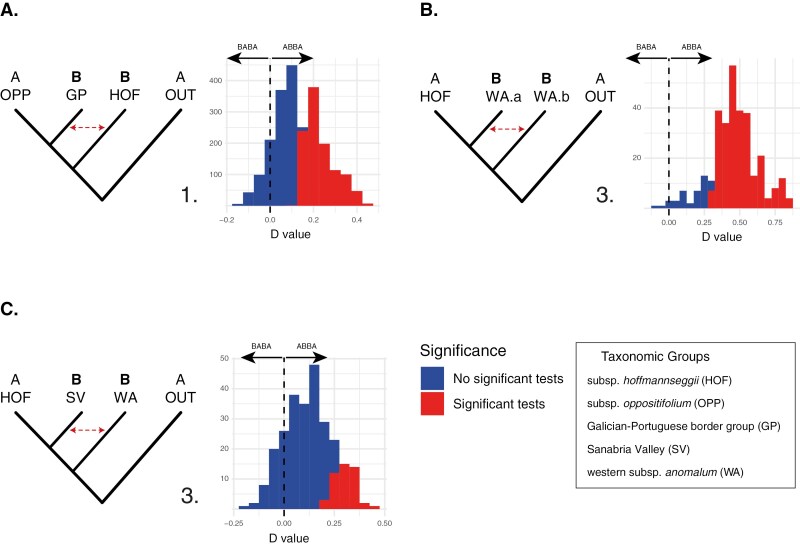
Summary of ‘D’ (ABBA–BABA) introgression tests for three of the hypotheses considered (only sets with the highest number of positive tests, A.2, C.3 and D.3, are shown): (1) the hybrid origin of the Southern Galician border group (GP) from hybridization between subsp. *hoffmannseggii* (HOF) and subsp. *oppositifolium* (OPP); (2) the different degrees of introgression in western subsp. *anomalum* (WA) populations from subsp. *hoffmannseggii* (HOF); and (3) hybridization in the Sanabria Lake between subsp. *anomalum* (WA) and subsp. *hoffmannseggii* (HOF). Dashed lines indicate introgression inferred from the tests. Height of the bars indicates the number of significant and non-significant individual tests. WA.a and WA.b denote different populations along the nuclear phylogenomic tree, resulting from different degrees of introgression of subsp. *hoffmannseggii*. The results of all tests are presented in Supplementary Data Table S4 and summarized in Fig. S2.

In the first set of scenarios, exploring potential introgression in subsp. *oppositifolium* or the southern Galician–Portuguese border group (GP) by HOF (A), all three provided significant positive tests of introgression, meaning that some populations of the tested taxa, not necessarily all, are likely to be introgressed (Fig. 4; Table 1; Supplementary Data Fig. S2; Table S3). However, the differences were profound depending on the subsp. *anomalum* group used (more positive cases when CA than when EA or WA was used as the outgroup). The tests also depended on the populations chosen as P1, P2 or P3, where, for example, no significant introgression was found with 5028 as P1 and 5038 as P2, but it was detected when 5040, a geographically close population, was used as P2. Similarly, introgression was detected with 4967/5044 as P3 and 5039 as P2, but not with 4729/4731 as P3 (Table S3). Taken together, 43.1 % of the individual tests in this set were significant, all showing an excess of the ABBA pattern indicating introgression between GP and HOF, consistent with HOF being a parent of GP. No significant BABA test was obtained to support HOF as the parent of OPP. In the second set of tests (B), which investigated the second parental lineage of GP and OPP by testing introgression between each of these groups and the subsp. *anomalum* groups, we obtained significant positive tests, all of which were consistent with the BABA pattern (Fig. 4; Table 1; Fig. S2; Table S3). Introgression between WA and HOF was supported by a considerable number of tests (B3, B6), whereas that between EA and HOF (B1, B4) and between CA and HOF (B2, B5) was not. In the third set of tests (C), which examined introgression into WA by other WA populations or other subsp. *anomalum* groups, the results were inconsistent. A strong signal was obtained using other populations of WA as P3 (83.33 %), with CA showing 22.22 %, and no introgression was detected using EA (Fig. 4; Table 1; Table S3). In the fourth set of tests (D), which examined introgression in populations in the Sanabria Valley (SV) populations by subsp. *anomalum* groups, positive significant tests were obtained when WA was used as P3 (16.79 %), and with a few cases when CA was used (3.06 %) (Fig. 4; Table 1; Table S3).

### Plastome phylogenomic analysis

A total of 129 samples were analysed: 121 *Phalacrocarpum* accessions from genome skimming and eight outgroups obtained from the NCBI SRA (Supplementary Data Appendix S1). After quality filtering of this entire dataset, we obtained an average of 1 202 126.403 reads (±681 296.9997 SD; 1378 min; 3 699 710 max) mapped to plastid targets (~2 % recovery). From these mapped reads, a median of 206 plastid regions (average 198, ±37 SD; 3 min; 209 max) were recovered with HybPiper at 50 % length. When considering only the 121 *Phalacrocarpum* accessions, the percentage recovery was the same, but the average number of reads mapping to plastid regions was higher (1 263 645.405 ± 641 579.316 SD; 251,196 min; 3 699 710 max), as was the average number of regions recovered with HybPiper at 50 % length (average 206, ±0.89 SD; 204 min; 208 max). One plastid region and five accessions (all outgroups) were discarded from the dataset based on their low overlap scores (less than two-thirds median coverage score). Seven other plastid regions were flagged as having potential paralogues (≥3 variants) and excluded (Appendix S2).

We generated 12 different datasets all containing 201 plastid regions depending on the threshold values set for the gap and the percentage of the original data matrix conserved. The proportion of parsimony-informative characters (PPIC) was 0.04 for all combinations (Table 3) and that for missing data was 1.26–0.46 (Table 3; Supplementary Data Appendix S3). Following ML-IQTREE analyses of the 12 datasets, we selected the gt0.1cons50 data matrix, i.e. the one in which the gap and conservation thresholds applied in trimAl were 0.1 and 50, respectively (Table 3). This matrix provided the best supported topology and was the most represented in the treespace landscape (results not shown). Although some differences were found among the ML trees generated from the 12 datasets, they mainly consisted in varying nodal support values for certain groups. The overall topology remained consistent, even among the most dissimilar trees, such as those obtained from the analyses of the gt0.1cons50 and gt09cons30 datasets.

**Table 3.** Alignment statistics across plastidial retrieved regions. *P*_PIC_, proportion of parsimony-informative characters.

Trimming parameter (trimAl)	Alignment length (bp)		*P*_PIC_ (%)		% Missing data	
	Mean	SD	Mean	SD	Mean	SD
gt01cons30	713.27	933.73	0.02	0.07	1.03	4.02
gt01cons50	714.24	933.39	0.02	0.02	1.19	5.14
gt01cons70	715.20	933.25	0.02	0.02	1.26	5.84
gt03cons30	710.75	932.16	0.02	0.02	0.70	3.12
gt03cons50	711.75	931.81	0.02	0.02	0.88	4.49
gt03cons70	713.07	931.48	0.02	0.02	1.04	5.48
gt07cons30	709.40	931.80	0.02	0.02	0.58	3.06
gt07cons50	710.40	931.44	0.02	0.02	0.76	4.45
gt07cons70	711.73	931.12	0.02	0.02	0.92	5.45
gt09cons30	706.12	931.00	0.02	0.02	0.46	2.79
gt09cons50	707.12	930.65	0.02	0.02	0.63	4.27
gt09cons70	708.59	930.24	0.02	0.02	0.82	5.31

**Fig. 5.**
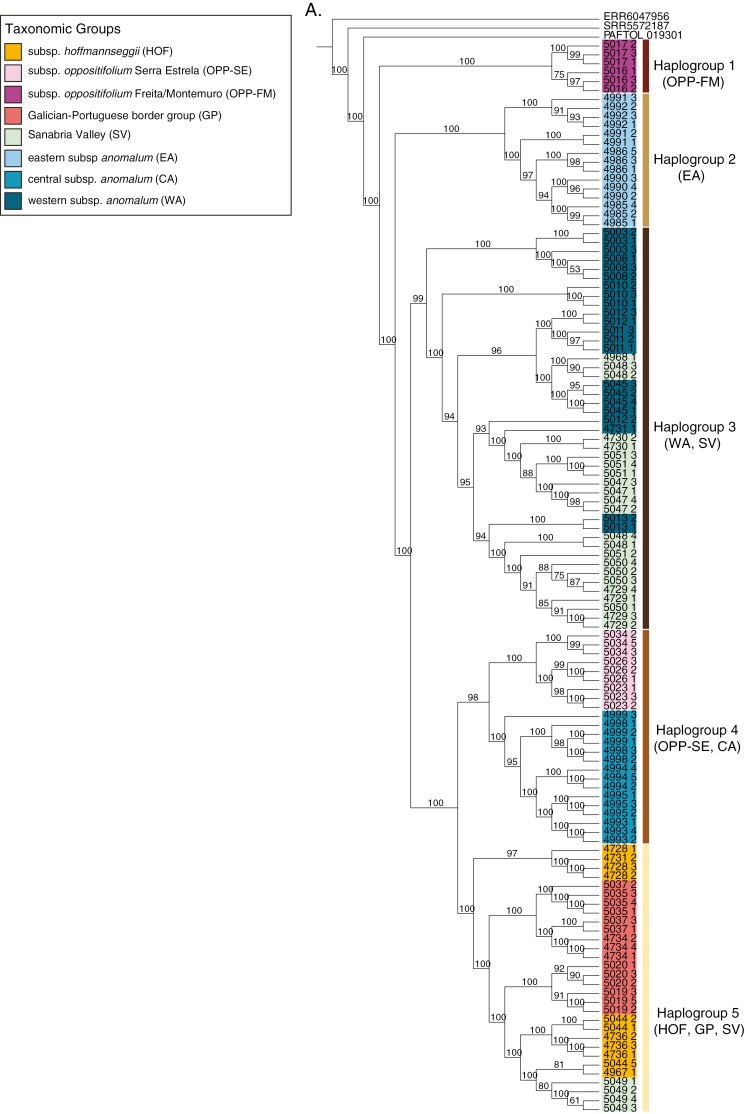
(A) Maximum-likelihood plastid tree of *Phalacrocarpum* based on complete plastomes assembled from genome skimming sequencing of 121 samples.

All *Phalacrocarpum* samples form a fully supported monophyletic group in the plastid tree (Fig. 5), but with discrepancies compared to the nuclear tree (Fig. 2). The initial split in the plastome tree leading to haplogroup 1 contains all the OPP-FM samples (populations 5016 and 5017). This fully supported clade is instead sister to the remaining subsp*. oppositifolium* samples (OPP-SE) in the nuclear tree (Fig. 2). The EA and CA populations do not form a monophyletic group. Instead, EA is the second fully supported group to diverge (haplogroup 2), whereas CA is sister to OPP-SE, forming a highly supported monophyletic group (haplogroup 4). This haplogroup is sister to a well-supported clade (haplogroup 5) that includes all HOF and GP samples together with those from one SV population (5049). The large fully supported clade encompassing haplogroups 4 and 5 is sister to another highly supported clade that includes WA populations and most of the SV samples (haplogroup 3) (Fig. 5).

## DISCUSSION

Some of the factors that complicate the understanding of hybridization scenarios are particularly relevant to our study (Abbott *et al.*, 2013; Meier *et al.*, 2017; Wagner, 2018). These include multiple hybridization events, either through continuous interspecific gene flow (Excoffier and Foll, 2011) or via episodes of recurrent but discontinuous gene flow (Princepe *et al.*, 2024), older hybridization events that may have led to the accumulation of genetic changes beyond the original genome merger, and population extinctions, including the extinction of parental taxa (ghost lineages), which may obscure the identification of hybrid lineages (Wolf *et al.*, 2001; Marques *et al.*, 2010; Tricou *et al.*, 2022). These phenomena may be connected to range dynamism, which plays an important role in facilitating contact between taxa and shaping ultimate outcomes such as introgression or plastid capture (García *et al.*, 2017; Liu *et al.*, 2020). In temperate regions, Pleistocene climatic changes were the main trigger for such dynamism (Hewitt, 2000;  Nieto Feliner, 2011; Sun *et al.*, 2014; Klein and Kadereit, 2016; Marques *et al.*, 2016). To address the effects of these interacting factors and understand the consequences of hybridization in *Phalacrocarpum*, an integration of different methodological approaches and sources of data is needed.

### Phalacrocarpum diversity patterns revisited

Our previous study (Criado-Ruiz *et al.*, 2021) reported three patterns that might have involved different evolutionary processes: a cryptic species that either arose through genetic drift or was an early divergent lineage in the genus, represented by eastern subsp. *anomalum* (EA); a probable hybrid lineage that underwent significant evolution after its hybrid origin in the Galician–Portuguese border (GP); and a more recent hybrid zone scenario in the Sanabria Valley involving HOF and WA. The integrated results from this study have provided insights that illuminate these processes.

**Fig. 6.**
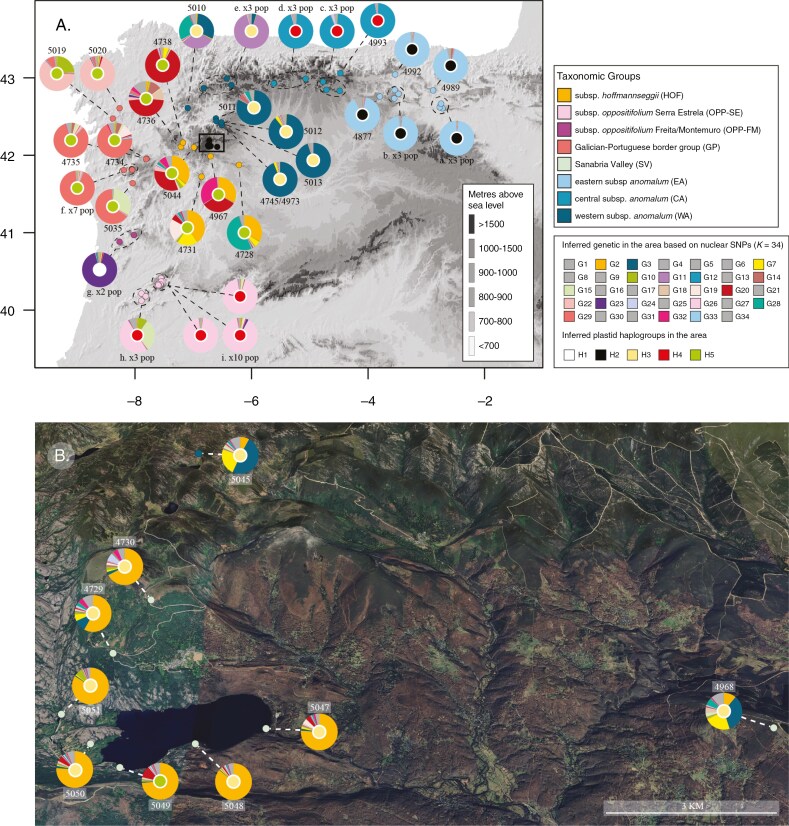
Genetic structure of *Phalacrocarpum* in a geographical context based on nuclear SNPs and full plastomes from 261 samples. The figure contains three colour codes, which are explained in the insets. Those in the dots directly on the map indicate the taxonomic groups described in the study. Pie charts show the nuclear ancestry inferred from SNPs by STRUCTURE as the mean posterior probability of assignment to each of the 34 genetic clusters (*K* = 34); groups with a posterior probability smaller that 0.1 are pooled into a grey code. Circles within the charts indicate the plastid haplogroup. Numbers next to the charts are the population codes of a single sampling point. In several cases, however, the chart represents a summary of the genetic structure in more than one sampling point (encircled by a dashed line): a (4985, 4986 and ALE2), b (4990, 4991 and ALE1), c (4994, 4995 and 4997), d (4998, 4999 and 5000), e (5003, 5004 and 5008), f (5036–5042), g (5016 and 5017), h (5032, 5033 and 5034), i (4976, 4977, 5023–5030). See Appendix S1 for sampling point information. (A) Whole distribution range. (B) Close-up of the Sanabria Valley, which includes Lake Sanabria and surrounding areas.

*(1) A cryptic species?* The rooted phylogenomic analyses of the nuclear and plastid sequences have allowed us to answer whether EA is a cryptic taxon or represents an early diverging lineage (Figs 2, 5 and 6). The notable genetic distance of EA from the remainder of the genus reflected in all analyses (see e.g. Figs 2, 3 and 5; Supplementary Data Fig. S3) is mainly due to the early divergence of this lineage and the apparent lack of secondary contact and hybridization with other groups. However, this study has unveiled the existence of a second genetic group within subsp. *anomalum* (CA) (Figs 2, 3 and 5). The relationships of CA in the plastome-based phylogeny, sister to OPP-SE, support the hypothesis that it was the maternal parent in a hybridization event that led to the homoploid hybrid lineage OPP-SE (see below), suggesting that CA underwent less geographical isolation than EA (Fig. 6).

*(2) Homoploid hybrid origin of GP and two OPP groups.* Our previous study, based on a low number of samples, found inconclusive evidence of hybridization underlying the evolutionary history of GP and, to a much lesser extent, OPP. For GP, the evidence here presented supports a single origin (Figs 2, 5 and 6) via hybridization between OPP and HOF (Fig. 4, Supplementary Data Fig. S2). STRUCTURE analysis also confirms the existence of significant genetic divergence after the hybridization event, which is thus inferred not to be recent. Such a trajectory probably involved a significant range expansion, as indicated by introgression into OPP-FM, HOF and WA (O Courel mountains) in the southern, eastern and northern edges, respectively (Figs 2 and 6).

For OPP, a distinct genetic group *c*. 80 km northwest of the core of this subspecies in the Serra da Estrela has been here identified (Serra da Freita, Serra de Montemuro; OPP-FM). This is apparent in all analyses, but the topology of the plastome-based tree is particularly intriguing since the plastome of these populations (haplogroup 1) is sister to the remainder of the genus (Fig. 5). According to our interpretation, this might reflect an old hybridization event between an expanding HOF and an early-diverging *anomalum* lineage that initially shaped the genetic composition of OPP-FM acting as maternal parent. Such a haplogroup 1 would have been retained in OPP-FM, but lost in subsp. *anomalum*. The current genomic composition of OPP-FM could have been subsequently shaped by both differentiation in isolation and secondary contact with GP as suggested by the G3 group (coral pink) in STRUCTURE (*K* = 7; Fig. 2). Compared to this newly discovered OPP-FM group, the core of subsp*. oppositifolium* (OPP-SE) appears to have undergone longer isolation, consistent with its geographical location at the southernmost edge of the genus range and the lack of admixture pattern (Fig. 2). However, support for the hybrid origin of OPP-SE – and also between subsp. *anomalum* and HOF – is provided by its phylogenetic position, nested within HOF in the nuclear tree, but sister to CA samples in the plastome-based tree. Based on the distinct plastomes of OPP-SE and OPP-FM, associated with two *anomalum* groups (CA vs. an early diverging plastome currently not found in *anomalum*, respectively), it is likely that hybridization events between HOF and *anomalum* were independent.

In summary, our evidence supports OPP-FM, OPP-SE and GP as three homoploid hybrid lineages within *Phalacrocarpum*. According to Nieto Feliner *et al.* (2017), these can be interpreted as hybrid species since they present signs of independent evolutionary trajectories visible in the phylogenomic trees and the Bayesian clustering analysis. Although two of them (GP, OPP-FM) have experienced gene flow with congeners after their formation, they are currently geographically isolated (Fig. 6).

*(3) The Sanabria Valley and recurrent hybridization in the crossroads.* With respect to the third pattern identified in Criado-Ruiz *et al.* (2021) involving the Sanabria Valley, the evidence gathered in this study suggests that hybridization events were more recent than those underlying the history of GP and OPP.

First, we consider the populations in the immediate vicinity of Lake Sanabria. This area is *c*. 3 km wide (Fig. 6). These were expected to represent recent products of hybridization based on their broad leaf morphological variability, albeit closer to HOF than to WA. The genetic results in our previous study were compatible with this expectation although the presence of a single Sanger sequencing-based plastid haplotype related to WA in that study was intriguing. However, the STRUCTURE analyses based on nuclear SNPs reveal that the ancestry of these populations predominantly corresponds to a genetic group (orange) shared with HOF, more evident at *K* = 7, although several other genetic groups are represented in very small proportions for *K* = 34 and *K* = 17. In contrast, traces of the WA genetic groups are nearly absent (Fig. 2; Supplementary Data Table S4). In the nuclear tree, these plants from the Sanabria Lake are intermingled with HOF, dispersed across different clades, most of which have low support and do not cluster together individuals from the same population (Fig. 2). In the plastid tree, all samples except those from one sampling site (see below) fall into a clade (haplogroup 3) that includes only SV and WA, confirming the hybrid origin of SV with WA as the predominant maternal parent (Figs 4 and 6). These findings suggest that although these populations surrounding the Lake underwent hybridization, it was probably followed by repeated backcrossing to HOF in the area and that the hybrid zone is not currently active, i.e. the second parent (WA) is not currently in the area and is not contributing gene flow to HOF or later generation hybrids or introgressants.

With respect to the occurrence only of a WA-like plastome, our study shows that although it is predominant (6 of 7 sampling points and 18 of 22 samples), the four samples of a single sampling point (5049) reveal that HOF also acted as maternal parent of hybrids in this area; this observation is consistent with a (former) hybrid zone. The predominance of the plastome of one of the parents (WA), despite backcrossing mainly occurring in the direction of the other parent (HOF), thus suggests the possibility of either cytonuclear incompatibility, selection or asymmetry in pollen–pistil interactions that result in reciprocal crosses not being equally viable (Briscoe Runquist *et al.*, 2014; Wang and Filatov, 2023).

The finding that the hybrid zone in Sanabria Valley is older than presumed may be more easily reconciled with the data if placed in a broader historical context. The inferred scenario points to a geographical area whose location fuelled the contact between northern (and higher-altitude) *anomalum* and southeastern (and lower-altitude) *hoffmannseggii*, at different times, which left traces in the form of introgression (Fig. 6). In fact, taking into account the geographical location of the Sanabria Valley, recurrent climatic changes forcing northward and southward range shifts in this genus, and its requirement for mountainous environments, may have made this valley and the adjacent areas (Montes de León) an inevitable crossroads.

Hybrid zones can be defined and subdivided in various ways (Jiggins and Mallet, 2000; Kawakami and Butlin, 2012), but the scenario in SV of a non-ongoing, or even old, situation does not fit the most classical notion of an ongoing hybrid zone where individuals from two populations meet and produce mixed offspring (Nieto Feliner *et al.*, 2023). Interestingly, as we move northwards and up in elevation away from the Lake, i.e. towards the WA niche, we sampled two populations (4968, 5045) that show a clearer admixture pattern in STRUCTURE (for the three *K* values) than the populations around the Lake and also exhibit the WA plastid haplogroup (Figs 2, 4 and 6; Supplementary Data Table S4). Morphologically, the first population is closer to HOF whereas the second is very similar to WA. They may represent more recent independent contacts between HOF and WA, which is consistent with a broader area for contact and hybridization between these two taxa beyond the Sanabria Lake.

Further north off the Sanabria Valley, WA populations from Montes de León (4745, 5011, 5012, 5013) form a well-supported monophyletic group, with a dominant dark blue (G4) genetic group, but also with several minor groups (*K* = 34 and *K* = 17) (WA3 in Figs 2 and 6; Supplementary Data Table S4). This admixture is consistent with this region being also part of a broad crossroads between WA and HOF. However, the signals of introgression are not restricted to SV and Montes de León, where it was more intense, but also reach nearby areas. The WA westernmost population sampled, from O Courel mountains (5010; WA2 in Fig. 2), display genetic groups for *K* = 7 that are shared with HOF, GP and OPP-FM (G1, G3 and G4). Based on the geographical proximity, it is plausible that this population had introgression from GP (Fig. 6). By contrast, the northernmost WA populations, located on the Cantabrian chain (5003, 5004, 5008; WA1 in Figs 2 and 6), only show one genetic group (G5), which suggests they underwent less introgression, or none at all, compared to the other two groups (WA2 and WA3).

### Proposed scenario for Phalacrocarpum evolutionary history

In this paper we present evidence of hybridization and introgression between different subspecies or genetic groups in *Phalacrocarpum*, namely between an early diverging *anomalum* lineage and HOF, CA and HOF, WA and HOF, HOF and OPP-SE, HOF and OPP-FM, GP and WA, and GP and HOF (Supplementary Data Fig. S5). Integrating the results from the different analyses, we infer that some of the hybridization events between the same two groups took place independently at different time periods, and some of them recurrently, in particular between HOF and subsp. *anomalum*. This is prominent in an area (Sanabria Valley, Montes de León) lying in the middle of an L-shaped chain of mountains that constitute the distribution range of this genus. Considering that the divergence of *Phalacrocarpum* from the rest of Leucanthemopsidinae is estimated to have occurred between 4.8 and 0.9 Mya (Criado-Ruiz *et al.*, 2024), the most likely scenario involved range shifts driven by Pleistocene climatic changes at different times mostly along and within this chain of mountains; this scenario is consistent with the cytonuclear disparities revealed here. These plants occur in small populations distributed in scattered humid mountain enclaves, often associated with outcrops. Years of cultivation in glasshouse conditions and outdoors have confirmed that they do not tolerate drought. It is thus likely that contraction and fragmentation of the distribution ranges of genetic groups was prompted by warm periods, as it is currently.

We propose two non-mutually exclusive explanations for the high number of genetic groups (*K* = 34) detected in STRUCTURE (Figs 2 and 6). The first is the effect of climate-driven shifts in range distribution patterns (expansion vs. contraction and fragmentation). Multiple contacts and hybridization events followed by fragmentation and drift may have led to populations with small effective population sizes and low levels of differentiation being identified by STRUCTURE as distinct genetic groups. The second possible explanation is likely to have contributed more to *K* = 34. This is related to the fact that the underlying model in STRUCTURE is not well suited to datasets containing clines of genetic variation (Guillot *et al.*, 2009; Pérez *et al.*, 2018) and tends to identify artificial clusters in these scenarios. Isolation by distance (IBD) can lead to clines of genetic variation (Wright, 1943). However, clinal variation can also result from hybrid zones (Barton and Heywitt, 1985). In *Phalacrocarpum*, clines of genetic variation from hybrid zones should have been produced not only in the Sanabria Valley, but probably also in the central parts of the genus range – the crossroads, including Montes de León – favoured by climate-driven range shifts. Some degree of IBD cannot be completely ruled out, especially for EA, which has evolved in isolation from other groups (Fig. 6). However, considering all the evidence, such as the admixture patterns in STRUCTURE, the introgression tests and the PCA plot, IBD cannot be the sole or main cause of clines of genetic variation. In fact, the entire nested scheme involving WA, HOF, GP, OPP-FM and OPP-SE, instead of these taxa each forming a monophyletic group, is best explained by hybridization, with WA and HOF acting as parental taxa, GP and OPP-SE as derivative hybrid taxa, and OPP-FM as both. Therefore, clinal variation resulting from hybridization seems to have been the main cause for an arbitrarily high number of genetic groups inferred by STRUCTURE. This would underscore the importance of considering suboptimal partitions of the dataset, as done here, where *K* = 7 is a good fit to the geographical and taxonomic patterns of diversity.

## CONCLUSIONS

Integrating the results from phylogenomics, Bayesian clustering analyses and introgression tests based on SNP data and full plastome sequences, we infer an evolutionary scenario for the Iberian endemic genus *Phalacrocarpum* that accommodates all molecular and ecogeographical evidence. Multiple (and partially recurrent) hybridization events occurring mainly along an L-shaped mountain corridor from the eastern Cantabrian range to the Serra da Estrela in central Portugal are inferred to have occurred, presumably during cold Pleistocene periods coinciding with range expansions. Some of these events led to three new homoploid hybrid lineages, which at least should be considered incipient hybrid species. Alternating with these cold periods, we hypothesize that warm periods fostered population range contraction and fragmentation, and genetic drift. This scenario involving a pronounced range dynamism can also explain the high number of genetic groups identified by Bayesian clustering analyses and the cytonuclear conflicts affecting in particular two plastid lineages (OPP-FM, OPP-SE).

A niche modelling study would be useful to determine how ecological factors may have contributed to the range expansion, survival and differentiation of some lineages, and to provide a more refined temporal framework for the hypothesized events. Understanding how biotic and abiotic factors interact in organisms where hybridization is important, such as *Phalacrocarpum*, may also contribute to our ability to predict future biodiversity patterns under global change.

## SUPPLEMENTARY DATA

Supplementary data are available at *Annals of Botany* online and consist of the following.

Fig. S1: Effects of the different *de novo* assemblies of the *Phalacrocarpum* ddRADseq data, based on two different parameters (clustering threshold, c; minimum number of samples, m), on various metrics. Fig. S2: Summary of ‘D’ (ABBA–BABA) introgression tests conducted in *Phalacrocarpum*. Fig. S3: NeighborNet diagrams showing relationships among 261 *Phalacrocarpum* samples based on SNPs using four different assemblies of the ddRADseq data. Fig. S4: NeighborNet diagrams showing relationships among subsets of the 261 samples of *Phalacrocarpum* based on SNPs, using the c90m131 assemby of the ddRADseq data. Fig. S5: Summary of hybridization and introgression events in the evolutionary history of *Phalacrocarpum* inferred from nuclear and plastid phylogenomic data. Table S1: Metrics obtained in *Phalacrocarpum* by processing ddRADseq data under different parameters for *de novo* assembly with Ipyrad. Table S2: Pooling of ‘populations’, i.e. sampling sites, into metapopulations used for calculating the maximum number of genetic groups (*K*) in the STRUCTURE analysis. Table S3: Results of the ABBA–BABA tests for different hypotheses showing the population accession used as P1, P2, P3 and P4. Table S4: Bayesian genomic clustering. Appendix S1: Samples used in this study for the ddRADseq and the genome skimming approach (GSA). Appendix 2: Recovery efficiency of HybPiper for target plastid regions studied. Appendix 3: Alignment summary statistics.

mcaf086_suppl_Supplementary_Figures_S1-S5_Tables_S1-S4
