# Supplementary material for: Homoploid hybrid speciation and recurrent hybridization along the northwestern Iberian mountain chains
Source: Ann Bot. 2025 May 5;136(2):325–42. doi: 10.1093/aob/mcaf086 (PMC12445855; doi:10.1093/aob/mcaf086)
Supplement: mcaf086_suppl_Supplementary_Figures_S1-S5_Tables_S1-S4 [file mcaf086_suppl_supplementary_figures_s1-s5_tables_s1-s4.zip › aob-24873-s01.docx]

Supplementary Material 1

***Selection of the RADSeq dataset assembly for subsequent analyses***

All BAPS analyses, using assemblies constructed with different parameters, identified five similar genetic groups (K = 5, data not shown). The results of the de novo assemblies using different parameters are shown in Supplementary Table S1. The c90m131 assembly was selected for subsequent analyses for the following reasons. This assembly produced a total of 1,699 loci including 43,441 variable sites, 27,936 of them parsimony informative, and 1,699 unlinked SNPs. This was one of the three best assemblies in terms of the number of loci, SNPs, and parsimony informative sites, and was selected over the other two based on the number of missing data and the absence of an outlier NeighborNet topology (Supplementary Figure S1; Supplementary Table S1).

***Results of the ddRADseq-based networks***

NeighborNet analyses based on unlinked SNPs identified between eleven and fifteen groups depending on the clustering threshold (c) used (Supplementary Figure S3). All networks exhibited a sort of neuron-like shape: two groups of subsp. *anomalum* (eastern and central populations, EA and CA, respectively) at one end (“the axon”) separated by a long axis from the remaining samples, which appear clustered at the other end (“the soma”). In the “soma” end, the network contains a number of parallel edges, indicating alternative connections and thus uncertainty. The western populations of subsp. *anomalum* (WA) are grouped together, but are connected by parallel edges with some of the Sanabria valley populations (SV). The southern Galician-Portuguese border populations (GP) are also grouped in almost all cases, with a few samples related to subsp. *hoffmannseggii* (HOF) and SV in c90m131 and c95m131 matrices. HOF samples are divided into two or three groups, as are SV samples, depending on the data matrix, intermingled with other taxa (WA, HOF, GP). *Phalacrocarpum* *oppositifolium* subsp. *oppositifolium* (OPP) is represented by two main groups which, depending on the pipeline, may or may not appear separated from each other. These are the populations of Serra da Estrela and surrounding areas (OPP-SE), and those from Serra da Freita and Serra de Montemuro (OPP-FM). The network resulting from the selected matrix (c90m131; see above) places the two OPP groups close to each other, unlike, e.g., c95m131 (Supplementary Figure S3).

In addition to the networks built from the whole data matrices, we constructed others in which some groups were removed. We first removed the most genetically distinct groups (EA and CA) to magnify the relationships among the remaining groups (Supplementary Figure S4 A). In the resulting network, there is a clear spread of SV samples, along with a relative separation of WA from other groups and a split of HOF into multiple axes. Removing SV provides a clearer separation of WA, OPP, GP and HOF (including some GP samples) (Supplementary Figure S4 B). These GP samples are not clustered within HOF when WA is removed from the network (Supplementary Figure S4 C). When analysing only the three subsp. *anomalum* groups and HOF, it becomes clear that although WA is morphologically indistinguishable from EA and CA, it is genetically more related to HOF (Supplementary Figure S4 D). When only WA and HOF are analysed, the WA group is not compact but scattered along three clusters (Supplementary Figure S4 E). Finally, including only SV, WA and HOF shows the SV samples spread along an axis that connects WA with HOF and that part of the WA samples are closer to HOF and SV than the rest (Supplementary Figure S4 F).
