# Supplementary material for: Homoploid hybrid speciation and recurrent hybridization along the northwestern Iberian mountain chains
Source: Ann Bot. 2025 May 5;136(2):325–42. doi: 10.1093/aob/mcaf086 (PMC12445855; doi:10.1093/aob/mcaf086)
Supplement: mcaf086_suppl_Supplementary_Figures_S1-S5_Tables_S1-S4 [file mcaf086_suppl_supplementary_figures_s1-s5_tables_s1-s4.zip › aob-24873-s04.pdf]

Gene tree reflecting no introgression

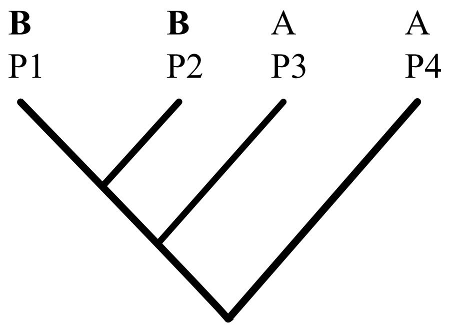

Introgression tests explored in *Phalacrocarpum*

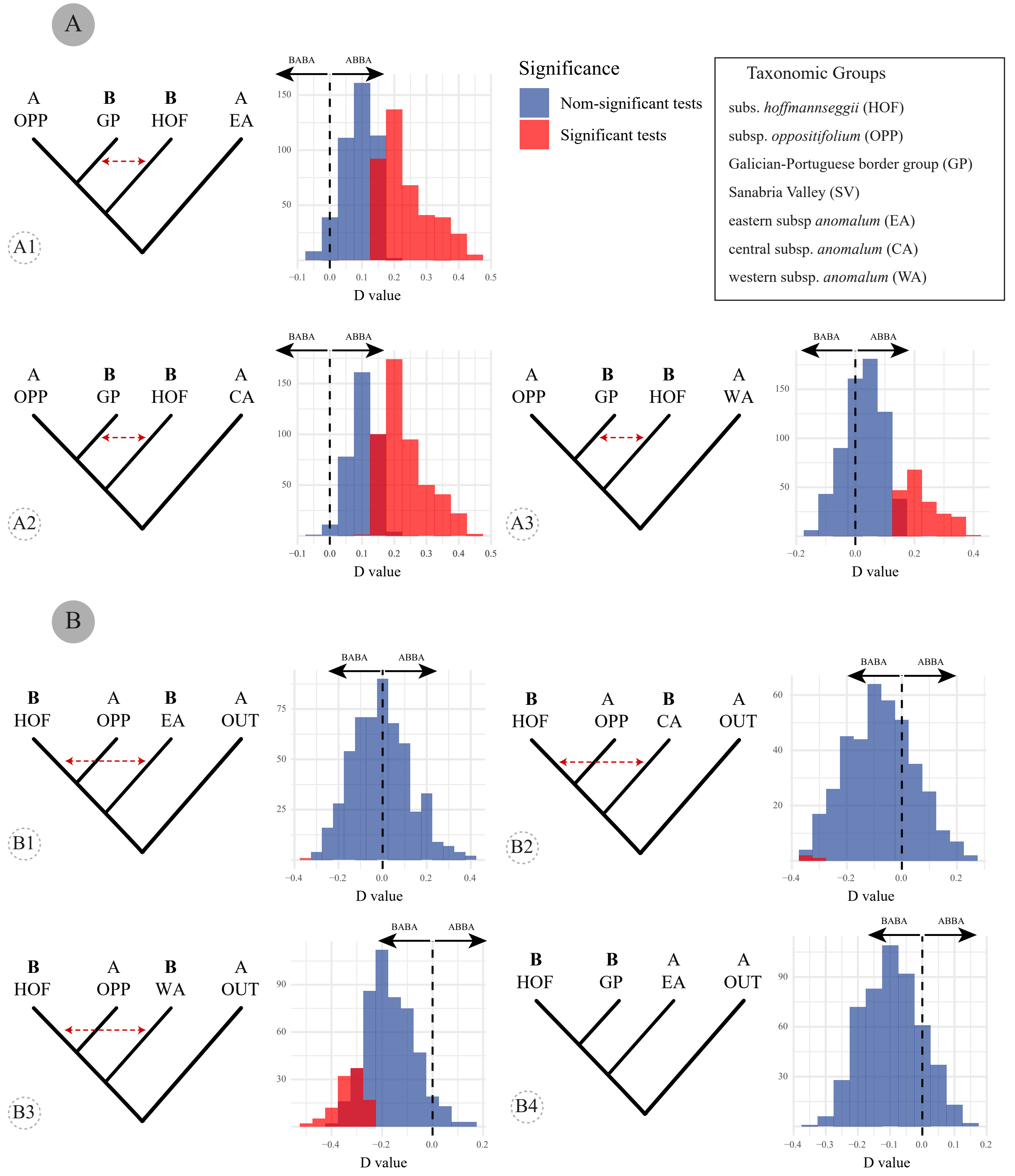

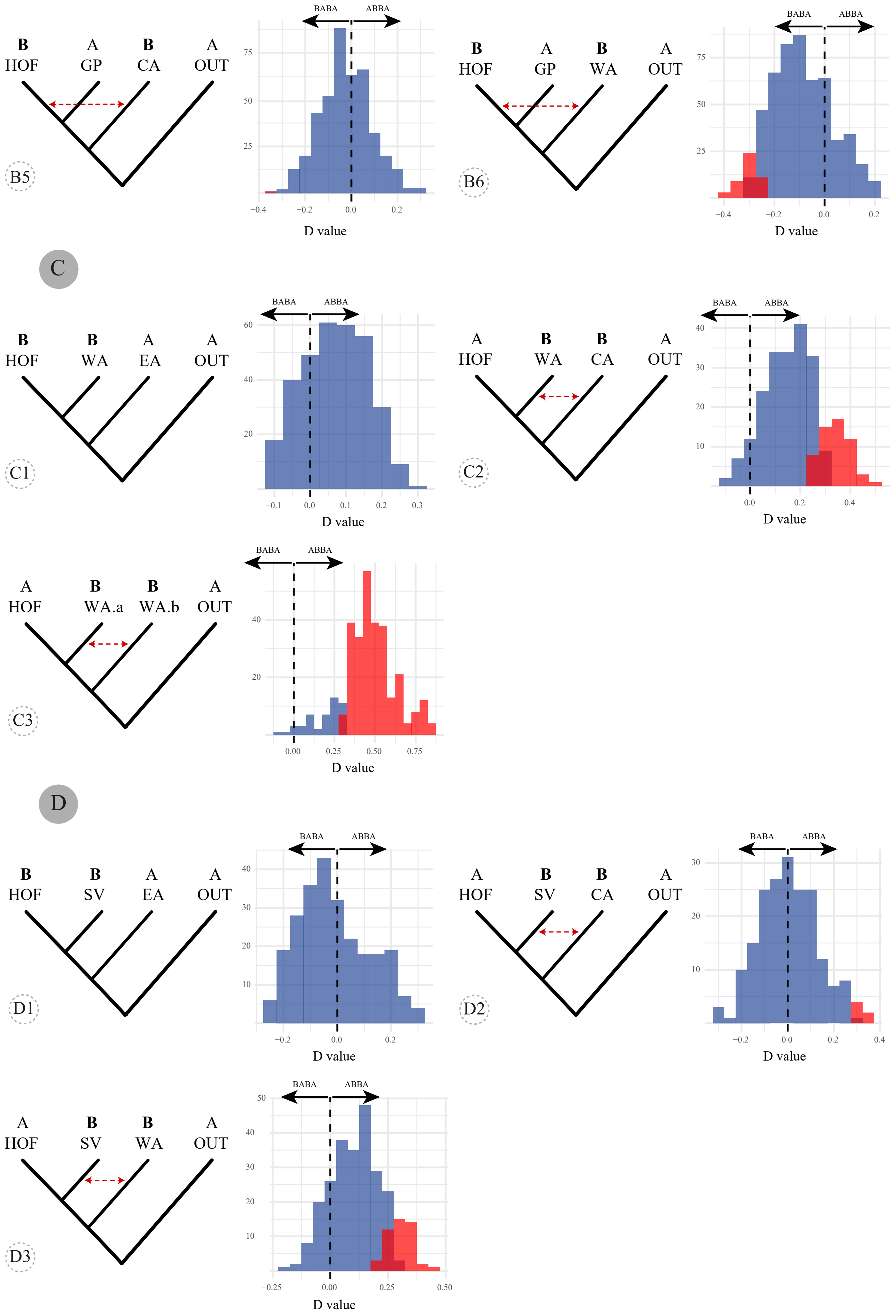

Figure S2. — Summary of "D" (ABBA-BABA) introgression tests conducted in *Phalacrocarpum*. For each hypothesis tested, the topological scheme is represented on the left, with dashed lines when introgression is confirmed. A bar diagram summarising the number of significant tests (in red) of the two patterns (ABBA, BABA) is shown on the right. WA.a and WA.b denote different populations along the nuclear phylogenomic tree, resulting from different degrees of introgression of subsp. *hoffmannseggii*.
