# Supplementary material for: Homoploid hybrid speciation and recurrent hybridization along the northwestern Iberian mountain chains
Source: Ann Bot. 2025 May 5;136(2):325–42. doi: 10.1093/aob/mcaf086 (PMC12445855; doi:10.1093/aob/mcaf086)
Supplement: mcaf086_suppl_Supplementary_Figures_S1-S5_Tables_S1-S4 [file mcaf086_suppl_supplementary_figures_s1-s5_tables_s1-s4.zip › aob-24873-s07.pdf]

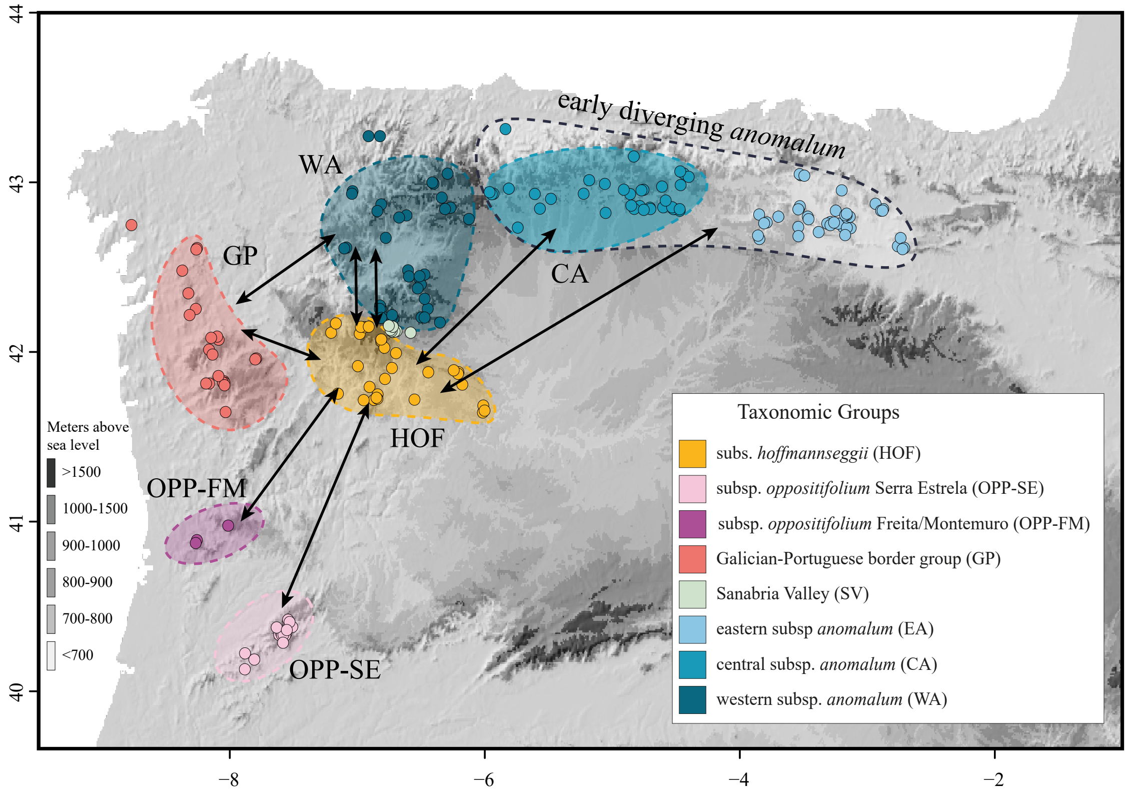

Figure S6. — Summary of hybridization and introgression events in the evolutionary history of *Phalacrocarpum* inferred from nuclear and plastid phylogenomic data. Arrows provide a flat representation (with no time component) of these events, with schematic ranges, i.e., roughly based on current ranges. See text.
