# Supplementary material for: Homoploid hybrid speciation and recurrent hybridization along the northwestern Iberian mountain chains
Source: Ann Bot. 2025 May 5;136(2):325–42. doi: 10.1093/aob/mcaf086 (PMC12445855; doi:10.1093/aob/mcaf086)
Supplement: mcaf086_suppl_Supplementary_Figures_S1-S5_Tables_S1-S4 [file mcaf086_suppl_supplementary_figures_s1-s5_tables_s1-s4.zip › aob-24873-s08.docx]

Supplementary Table S1. Metrics obtained in *Phalacrocarpum* by processing ddRADseq data under different parameters for de novo assembly with Ipyrad. The differences are based on the c value (clustering threshold above which two fragments are considered to belong to the same locus) and the m value (minimum number of samples with a given locus to accept it). The final selected assembly used in subsequent analyses is highlighted in grey. The bottom row shows the metrics for the selected assembly plus the outgroups used in the phylogenomic analysis with PoMo. *PSI: parsimony informative sites.

| De novo assembly | Samples | Loci | % Missing data sites | Total SNPs | PSI | Unlinked SNPs |
| --- | --- | --- | --- | --- | --- | --- |
| c85m131 | 261 | 1442 | 22 | 36443 | 23559 | 1441 |
| c88m131 | 261 | 1581 | 22.32 | 39841 | 25768 | 1580 |
| c90m131 | 261 | 1699 | 22.45 | 43441 | 27936 | 1699 |
| c95m131 | 261 | 1847 | 22.56 | 43893 | 27838 | 1847 |
| c90m78 | 261 | 2600 | 34.99 | 63271 | 39851 | 2600 |
| c90m183 | 261 | 1122 | 13.82 | 28605 | 18745 | 1122 |
| c90m222 | 261 | 663 | 7.89 | 16590 | 11049 | 663 |
|  |  |  |  |  |  |  |
| c90m131 with OUT | 265 | 1597 | 23.76 | 45801 | 27222 | 1597 |
