# Supplementary figures and images for: Homoploid hybrid speciation and recurrent hybridization along the northwestern Iberian mountain chains
Source: Ann Bot. 2025 May 5;136(2):325–42. doi: 10.1093/aob/mcaf086 (PMC12445855; doi:10.1093/aob/mcaf086)

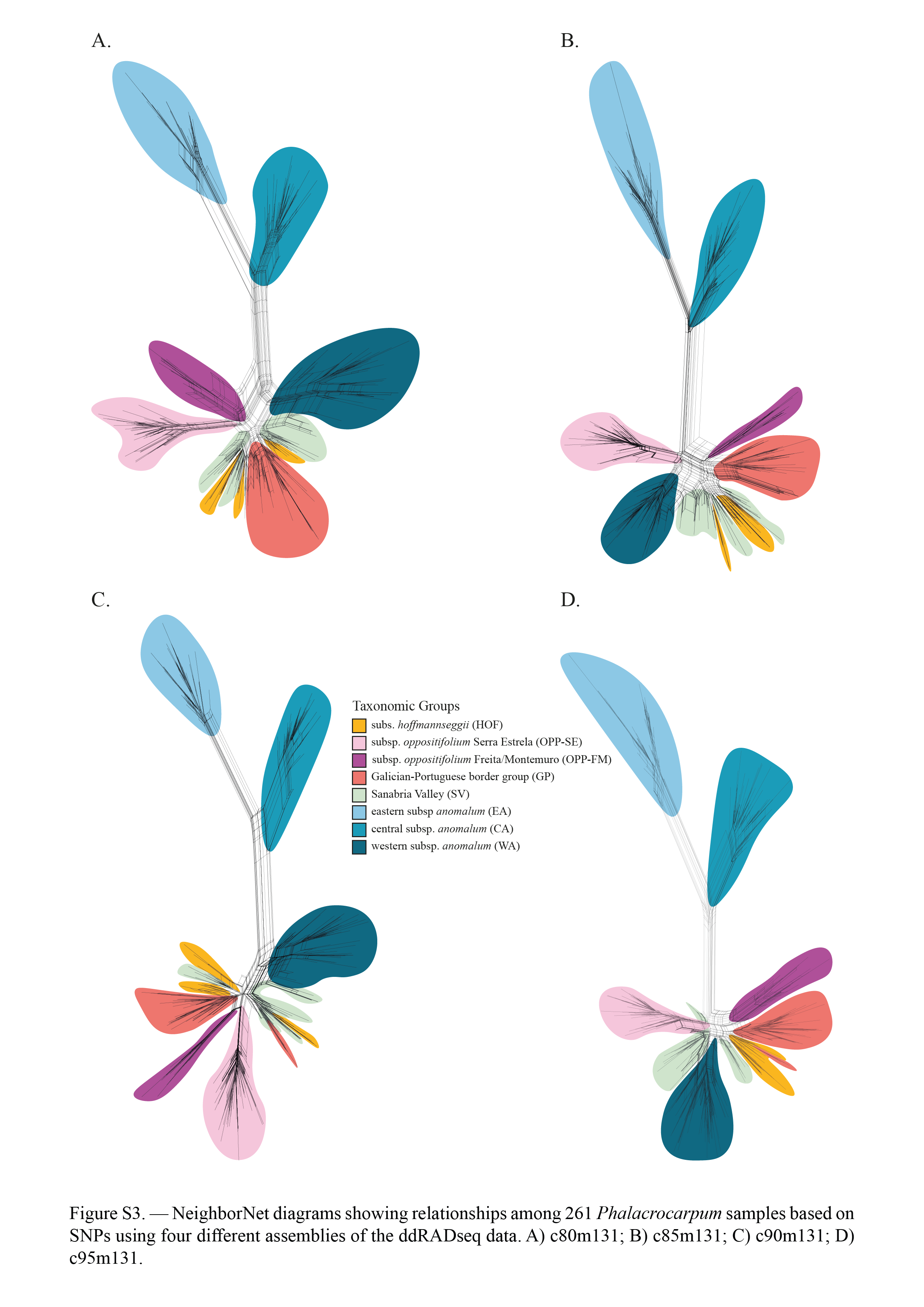

Supplement: mcaf086_suppl_Supplementary_Figures_S1-S5_Tables_S1-S4 [file mcaf086_suppl_supplementary_figures_s1-s5_tables_s1-s4.zip › aob-24873-s05.png]

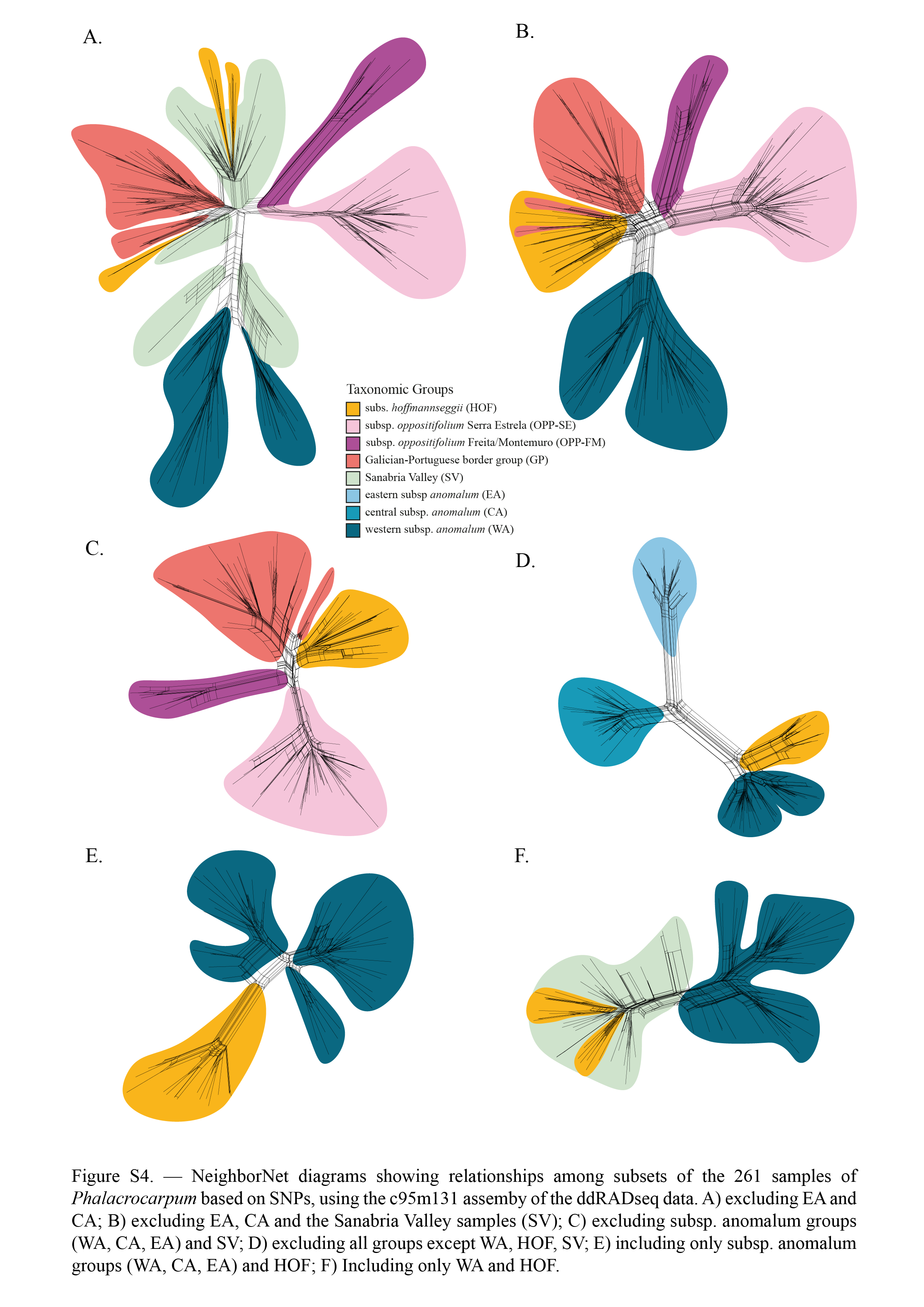

Supplement: mcaf086_suppl_Supplementary_Figures_S1-S5_Tables_S1-S4 [file mcaf086_suppl_supplementary_figures_s1-s5_tables_s1-s4.zip › aob-24873-s06.png]
