# Supplementary material for: Homoploid hybrid speciation and recurrent hybridization along the northwestern Iberian mountain chains
Source: Ann Bot. 2025 May 5;136(2):325–42. doi: 10.1093/aob/mcaf086 (PMC12445855; doi:10.1093/aob/mcaf086)
Supplement: mcaf086_suppl_Supplementary_Figures_S1-S5_Tables_S1-S4 [file mcaf086_suppl_supplementary_figures_s1-s5_tables_s1-s4.zip › aob-24873-s02.docx]

*Abidjan, Côte d’Ivoire*

*April, 10^th^ 2025*

***Rowan Sage
Chief Editor
Annals of Botany***

**Covering letter for** **submission of the revised version of manuscript** **25072 in Annals of Botany**:

*A spatial perspective on flowering in cauliflorous cacao: architecture defines flower cushion location, not its early activity*

*by* T. Wibaux, P.É. Lauri, A.A. M’Bo Kacou, O. Pondo Kouakou and R. Vezy

Dear Editor,

We would like to thank the editorial board for their positive assessment of our article. We are also grateful to the handling editor and the two reviewers for their careful reading of our manuscript and for their valuable comments, which helped improve the clarity and content of the paper.

Below, you will find a detailed list of the changes made to the manuscript, along with our point-by-point responses to the reviewers' comments. Comments from each reviewer appear in italic and are preceded, when relevant, by the corresponding page and line numbers of concerned section in the 1^st^ submission’s manuscript. Each comment is followed by our response. We hope that these revisions and answers to the reviewers’ comments will clarify our objectives and strengthen the overall contribution of the manuscript.

To facilitate the review of our revisions, we uploaded a version of the manuscript with tracked changes as supplementary material during the submission process (not to be published).

Yours Sincerely,

*Thomas WIBAUX,*

*CIRAD, UMR ABSys*

*Université Félix Houphouët-Boigny, CEA-CCBAD/WASCAL*

*Pôle Scientifique et d’Innovation, Bingerville*

*CIRAD, 01 BP 6483 Abidjan, Côte d’Ivoire*

*Cel : (+225) 07 89 65 22 18*

*Email : thomas.wibaux@cirad.fr*

**Response to the Handling Editor's** **comments:**

We kindly thank the handling Editor for his appraisal of our work and his positive review of our manuscript. In answer to the suggestions that he provided, we made the following arrangements to the manuscript:

- A glossary table was added in the core text (Table 1), which includes definitions of the architectural and morphological terms most specific to cacao and this study. We also adapted the abstract to make it easier to understand for a broad public, using the most common terms used for describing cacao architecture (node, flush, shoot).
- The details of the structure and outputs of the models performed for the study of architectural and geometrical factors on each flowering variable were added as supplementary material, in an appendix (see our response to the corresponding comment from referee #2).
- We added the missing letters on multi-panels figures and reformatted the figures to fit expected dimensions and quality for publishing.
- We improved references to corresponding figures, tables and appendices in the discussion.
- We increased the upper limit of the y-axis on the top panel of the last figure, for better readability.

**Response to the comments of referee #1:**

General comments:

- *“First, the introduction is very detailed giving an in-depth description of cauliflory and cocoa flowering without clearly indicating what the unknowns are and why it is so important to address these. Second, the paper will probably be mostly read by the increasingly large number of people working on cocoa (…) I would turn the introduction around starting with cocoa and then work towards its flowering. Third, the discussions is lacking in addressing the question what these results could mean for management and maybe prediction of cocoa production”.*

The authors agree that cacao cultivation practitioners expect practical guidelines to address the yield gap associated with cacao flowering or improve yield prediction based on flowering monitoring. However, this article intentionally focuses on cacao as a model species to study cauliflorous flowering, with the primary aim of advancing fundamental knowledge on its reproductive functioning in light of this unique trait. This focus on fundamental aspects of cacao biology is also the reason why we chose to submit the manuscript to a botanical journal. Other findings from the same study, that address agronomic applications and management practices, have been (or are being) published in agronomy journals. We hope that this clarification also responds to the reviewer’s comments concerning the closure of the paper.

Abstract

- 3.4 *“It comes as a bit of dissapointment to read that this research was conducted on juvenile trees. The part I do not understand is, what is the point of flowering to a tree if it is not producing fruits”:*

We appreciate the reviewer’s concern about using young, unproductive trees. Our main objective was to understand the conditions leading to flower cushion establishment at individual nodes, thus focusing on first flowering on the node i.e., the initial appearance of flower cushions. Although first flowering of a node can be observed on young axes of productive trees, flowers and fruits are mostly borne on older axes (1st to 3rd order), established early in the tree’s life. Studying young trees thus helped us capture the conditions of floral development on the parts that ultimately bear most of the yield. More broadly, we believe flowering should often be studied separately from fruiting, to avoid confounding factors such as pollination success or resource limitations that affect fruit set and development.

- 3.5-6: *“ending a paper with "we hypothesize" at least would not be my style.”*

We recognize that this phrasing may appear unusual in the context of an abstract. The same comment could be made on the last sentence “we highlight the need for further analysis”. The conclusion of the abstract was changed to be more assertive (Lines 23-28 in revised document).

Introduction

- *“Overall the introduction is very elaborate on the architecture of cocoa. I was wondering whether all this description is needed. At the same time the problem statement section is very short. This makes it difficult to determine what the exact knowledge gap is that the authors try to address and why it is important to address it.”*

We agree that this section is quite exhaustive, but we feel that it is necessary to properly describe the specificities of cacao architecture to introduce our study on architectural determinisms in flowering. Also, this article is the first over the last 30 years to address the subject of tree architecture of *Theobroma cacao*. We felt it was timely to take advantage of the publication of an article on cacao tree growth and architecture in a botanical journal to provide a comprehensive overview of the existing knowledge on the subject. This is especially relevant since this information is scarce and often quite old, difficult to access, or even inaccessible to most (published in French, not indexed online, etc.). Although we opted for a quite extensive presentation of the context and available knowledge in this introduction, we also explicitly state the specific knowledge gaps that will be addressed in this article in lines 110 – 114.

*Materials & Methods*

- *“It is unclear what is meant with the term "genotype" here.* *Are these the normal " hybrids" obtained from the national breeding programs. These are usually not very well defined.” (…) “It is hard to determine how many replicate trees there were per shade treatment by genotype combination”. (…) “It says "nine selected trees". How do these relate to the earlier mentioned 24 trees?”*

We acknowledge that this section lacked clarity and was confusing. In answer to the comment, we revised this section to avoid any confusion and explain with more details the origin of studied genotypes (lines 128-134) and the experimental design and number of replicates per genotype and shade treatment (lines 145-150).

- 11.8 *“I think it is very interesting to look at flowering synchronicity, but the introduction does not explain why the authors determined this.”*

The question of presumed synchrony versus potential asynchrony in the flowering dynamics of flower cushions at the tree, crown, or trunk scale is introduced in lines 98-110. In response to this comment, we have clarified and expanded the explanation of the knowledge gaps surrounding this issue in lines 110-112, to better justify the focus on flowering synchrony.

Results

- 13.16 *“The results section starts with mentioning the number of phytomers that were analyzed. These need to be nested in trees for statistical analyses. That is, two phytomers from the same tree are not independent and this needs to be accounted for”*

We thank the reviewer for this relevant remark. We fully agree that phytomers borne on the same tree cannot be considered independent. In our study, we analyzed a single branch per tree, and each branch was assigned a unique identifier (“Branch Id”), which thus corresponds to a unique tree. We accounted for the non-independence of phytomers within trees by including “Branch Id” as a random effect in all statistical models. This approach explicitly addresses the nested structure of the data. In answer to the comment, we adjusted the corresponding paragraph in M&M to explicit with more clarity this consideration of nested effects (lines 253-257).

- 17.10 *“Better to say heavy shade than high shade.”*

We apology for this error, high shade has indeed an ambiguous meaning (shade intensity VS height of the shade stratum). We changed the expression to “heavy shade” throughout the document and in the figures.

**Responses to the comments of Referee #2**

Materials and methods

- *“Plant materials and experimental design- Suggest providing environmental conditions throughout the years in brief and also coordinate the location.”*

We thank the reviewer for this insightful comment, which highlighted an important omission of crop management information in our description of the experimental conditions. Specifically, the trial was conducted with supplemental irrigation to mitigate the effects of the pronounced climatic seasonality characteristic of the study region. We added this key information in the M&M section, along with information about fertilization and a reference to more detailed information about climate and crop phenology (lines 141-145). The GPS coordinates of the plantation site were also added in line 127.

- (8. 10) *“provide how you measured light intensity.”*

The references of PAR sensors used in the study were added to the manuscript (lines 138 - 140).

- *“The authors mention "architectural and geometrical measurements, but they could be more specific about the exact measurements taken at each level (phytomer, growth unit, axis). (…) How was the "presence of floral buds or flowers at anthesis" assessed? Was it a visual inspection?”*

The section “Branch, axis and growth unit architecture and geometry” (lines 154-190) provides a detailed description of each measurement performed at the axis, growth unit, and phytomer levels. In addition, Table 2 presents the complete list of observations and measurements collected in this study, including the type of variable, modalities and scales of measurement. In response to the reviewer’s question we have clarified in the manuscript that all data were collected through visual observations, complemented by hand markings and direct measurements on the trees (lines 155-157 and line 194-195)

- *“The Synchrony Index can be explained in more detail.”*

We adapted the paragraph of M&M on the calculation of the Synchrony index to add details on our use of Augspurger’s Index (lines 216-221), and the way to interpret the value of the index (lines 239-241).

Results and discussion

- *“While the authors mention using GLMM and stepwise modeling, they could be more explicit about the final model structures”; “Presenting the full model outputs (e.g., coefficients, standard errors, p-values) in tables would also be beneficial”*

We thank the reviewer for this suggestion. For conciseness, we chose to present only the full model outputs for the model that showed significant effects of architectural or geometrical factors, and only provided a summary of the final model results for other studied variables. However, we agree that providing the complete outputs of all final models, regardless of significance, will improve the transparency and reproducibility of our analyses. To avoid overcrowding the manuscript, we propose to add in an appendix the detailed structure and results of models with best-fit yielded by the stepwise approaches, for each studied variable. We hope this addresses the reviewer’s concern.

- *“Selecting only one second-order branch per tree and including a limited number of trees in the study might limit the ability to draw broader conclusions about flower cushion formation and activity in cacao. Have you considered the potential for variation among branches within a tree and across a larger population of trees?”*

This is a relevant comment regarding our methodological choices. Our priority was to conduct observations of flower cushions at the phytomer scale, to explore their potential functional independence or coordination. Lacking prior references on cushion location, timing of establishment and functioning, we opted to avoid sampling and monitor all positions on the trees. However, we underestimated the trees' growth and the volume of data required. To address this, we chose to focus on a subset of branches on each studied tree (a unique secondary branch) rather than reduce the number of trees. This strategy allowed us to analyze cushion functioning at the growth unit, axis, and branch scales while maintaining sufficient replication to detect genotype and shading effects. We acknowledge that working on a small number of trees and genotypes limits broader conclusions on species-level phenotypic variation or breeding applications. But we believe that this approach was the most appropriate for our objectives. Nonetheless, the extensive dataset allowed robust analyses and the detection of significant experimental effects. It also provides a basis for rational, architecturally justified, sampling strategies in future studies, allowing work to be carried out on larger tree populations.

- *“What are the implications of the observed differences between genotypes?”; “The authors need to discuss genotypic differences between the two genotypes used in this study and how genetics impacted the physiological and molecular aspects of the cacao branch and flower.”*

In response to the reviewer’s question, we would like to clarify that studying only two genotypes will not be sufficient to address, or even discuss, the genetic-dependent molecular or physiological mechanisms underlying the observed differences. This is especially true since cauliflory is an unusual trait for which there are few existing studies or references to build upon. Our comparison aimed at testing for possible differences between two contrasting genotypes, to reveal potential genetic influences on flower cushion establishment and flowering dynamics. The significant differences that we observed between the two genotypes, in the establishment of flower cushions and in the duration and frequency of floral episodes, support the hypothesis of genetic control over these traits but it is not sufficient to address thoroughly the physiological and molecular mechanisms involved. However, our findings provide a basis for future studies specifically aimed at investigating the genetic and studying such mechanisms. We added a sentence reflecting this reasoning in the relevant section of the discussion (lines 411-415), in response to the reviewer’s comment.
